# Elevated Pressure Effects on Plasma-Driven Ammonia Synthesis: Insights from Experiments and Kinetic Modeling

**DOI:** 10.1021/acssuschemeng.5c06251

**Published:** 2025-09-11

**Authors:** Jintao Sun, Weitao Wang, Chunqiang Lu, Xin Tu

**Affiliations:** Department of Electrical Engineering and Electronics, 4591 University of Liverpool, Liverpool L69 3GJ, U.K.

**Keywords:** ammonia synthesis, nonthermal plasma, elevated pressure, kinetic modeling, reaction pathways

## Abstract

Nonthermal plasma (NTP) presents a promising pathway for sustainable ammonia synthesis under mild conditions, enabling activation of nitrogen without the need for high thermal input. While most studies to date have focused on plasma ammonia synthesis under ambient pressure, the potential benefits of elevated pressure, such as improved thermodynamic favorability and enhanced compatibility with downstream ammonia separation technologies, remain underexplored. In this work, we investigate plasma-driven ammonia synthesis under elevated pressure by combining experimental measurements with detailed plasma chemical kinetics modeling. A dielectric barrier discharge plasma reactor was employed, with the system pressure controlled up to 3 bar using a high-pressure regulator. Contrary to thermodynamic expectations, the experimental results reveal that increasing pressure suppresses ammonia yield in the plasma environment, primarily due to a reduction in the reduced electric field (E/N), which diminishes the energy of electrons available for molecule activation. The underlying reaction mechanism was elucidated using in situ optical diagnostics and chemical kinetics simulations. Path flux analysis confirms that N_2_ is dissociated by energetic electrons into N and excited N­(^2^D) species, which are subsequently hydrogenated to form NH and NH_2_ radicals. These intermediates recombine via NH_2_ + H­(+M) → NH_3_(+M) and NH + H_2_ + M → NH_3_ + M to form ammonia. Notably, elevated pressure does not alter the dominant reaction pathways but significantly influences the reaction rates and plasma characteristics. Sensitivity analysis highlights that the electron-impact dissociation of N_2_ [e + N_2_ → e + N + N­(^2^D)] is the rate-limiting step and has the greatest promoting effect on ammonia formation. These insights offer guidance for optimizing plasma operating conditions and advancing the practical application of plasma-assisted ammonia synthesis under pressurized conditions.

## Graphical abstract

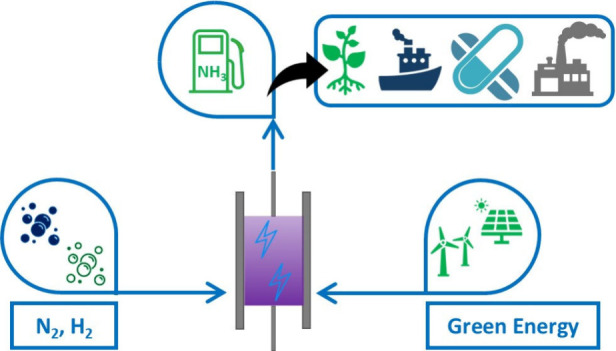

## Introduction

Ammonia (NH_3_) is a fundamental chemical in the modern chemical industry, serving not only as a key precursor for nitrogen fertilizer production essential to global food security, but also as a promising carbon-free energy carrier for hydrogen storage, transportation, and fuel applications.^1,2^ The Haber–Bosch (HB) process, developed in the early 20th century, remains the dominant route for industrial ammonia synthesis. This process relies on the reaction (N_2_ + 3H_2_ ⇋ 2NH_3_; Δ*H*_298 K_ = −46.35 kJ/mol) between nitrogen (N_2_) and hydrogen (H_2_) over an iron-based catalyst under harsh reaction conditions (450–550 °C, 200–300 bar).^3,4^ High temperatures are required to break the strong N≡N bond (945 kJ/mol) of the N_2_ molecule and drive the reaction. However, low temperatures favor ammonia production due to the exothermic nature of this reaction. While highly efficient and technologically mature, this process faces significant challenges, including intensive energy consumption (30 GJ/t_NH_3__) and substantial CO_2_ emissions (2 kg_CO_2__/kg_NH_3__), accounting for approximately 1–2% of global greenhouse gas emissions.^5–7^ Furthermore, its dependence on centralized, large-scale infrastructure limits its adaptability for flexible and decentralized ammonia production using intermittent and distributed renewable energy sources such as wind and solar power.^8,9^ In light of these challenges, increasing attention has been directed toward alternative low-carbon strategies for ammonia synthesis via N_2_ activation, such as electrocatalysis,^10^ photocatalysis,^11^ and plasma-driven synthesis.^1,12^ These approaches can in principle activate reactants under comparatively mild conditions. In electrocatalytic N_2_ reduction (eNRR), ammonia formation typically proceeds through the stepwise reduction of adsorbed nitrogen intermediates, which generally requires a relatively high overpotential. However, the kinetically favored hydrogen evolution reaction inevitably competes with N_2_ reduction, consuming a substantial fraction of electrons and protons and thereby resulting in low Faradaic efficiency and energy utilization. In photocatalysis, N_2_ activation is further constrained by the intrinsically low photon utilization efficiency and rapid recombination of photogenerated charge carriers, leading to limited NH_3_ synthesis rates under practical conditions. Nonthermal plasma (NTP) provides a distinct approach by generating a partially ionized gas containing a mixture of energetic electrons and reactive species (e.g., radicals, ions, and excited species), while maintaining a low bulk gas temperature. This unique character enables N_2_ activation and ammonia synthesis under mild conditions (e.g., room temperature and ambient pressure).^13,14^ Energetic electrons in the plasma effectively activate the N≡N bond via vibrational and electronic excitation, reducing the reaction energy barrier and avoiding the proton-coupled electron-transfer limitations encountered in eNRR. Compared to photocatalysis, NTP sustains a high density of reactive species, enabling more efficient and stable ammonia formation with enhanced reaction rates and productivities. Plasma reactors also offer rapid start-up and shut-down, making them highly compatible with intermittent renewable electricity and suitable for decentralized production.^15^ Importantly, plasma systems typically do not require expensive electrode or materials, and even when coupled with catalysts, effective activity can be achieved using earth-abundant transition metals or oxides rather than costly noble metals. These characteristics make NTP a particularly promising flexible electrification solution for sustainable ammonia synthesis under mild conditions.

Currently, various NTP sources have been developed for ammonia synthesis, including gliding arc,^16^ dielectric barrier discharge (DBD),^17^ radio-frequency discharge,^18^ microwave discharge,^19^ and glow discharge.^20^ DBD has attracted particular attention in plasma-based chemical transformation (e.g., ammonia synthesis, CO_2_ hydrogenation, and methane conversion) due to its notable advantages, including scalability, strong compatibility with catalysts, and stable operational performance.^21–23^ Most previous works have focused on improving the performance of the plasma ammonia synthesis process by tuning plasma parameters, modifying reactor configurations, or integrating different catalysts. It has been reported that the best energy yield of ammonia synthesis from an N_2_/H_2_ mixture using plasma alone is 7.7 g_NH_3__/kWh,^17^ whereas plasma-catalytic synthesis can achieve up to 36.0 g_NH_3__/kWh.^24^ A thorough understanding of the reaction mechanism is important for improving process performance. The current consensus is that in an N_2_/H_2_ plasma, both N_2_ and H_2_ can be activated and dissociated into various reactive species.^25^ Simek et al. emphasized the importance of the metastable state of N_2_ [N_2_(A^3^)], due to its relatively high energy level (6.2 eV) and long lifetime (10^–5^–1 s).^26^ NH radicals are considered key intermediates in ammonia synthesis, primarily formed through the recombination of N and H atoms, although they may also be generated via reactions between N* and H_2_ or between N_2_^+^ and H.^27,28^ These NH radicals can undergo subsequent hydrogenation to form NH_2_ radicals, and, ultimately, ammonia is produced through further hydrogenation of NH_2_ and NH species. However, the formation of NH and NH_2_ radicals is influenced by various factors, including competing reactions. Furthermore, energetic electrons in the plasma can decompose the formed ammonia through dissociation (e + NH_3_ → e + NH_2_ + H; e + NH_3_ → e + NH + H_2_) and ionization (e + NH_3_ → 2e + NH_3_^+^) to form NH_*x*_, H, and other intermediates, leading to a reduction in ammonia yield and energy efficiency.

Most current research on plasma-based ammonia synthesis has been conducted at ambient pressure. However, operating at elevated pressure offers thermodynamic and process integration advantages that are currently underexplored. According to Le Chatelier’s principle, increasing pressure favors ammonia formation in this exothermic reaction, potentially improving conversion. Furthermore, operating under moderate to high pressures can significantly enhance compatibility with downstream separation technologies, such as pressure-swing adsorption, which are commonly employed for ammonia separation. These processes are typically more energy-efficient when coupled with upstream systems operating at elevated pressure, as they avoid the need for costly compression steps postsynthesis. Therefore, pressure not only impacts reaction equilibrium but also plays a critical role in overall process efficiency and system integration, particularly in decentralized or modular ammonia production systems. Despite these compelling advantages, the fundamental understanding of plasma-driven ammonia synthesis under elevated pressures remains limited. This is partly due to the experimental complexity and plasma instabilities associated with high-pressure operation. To address this gap, we investigate an NTP-driven ammonia synthesis from N_2_ and H_2_ at pressures up to 3 bar. Through a combination of in situ optical diagnostics and plasma chemical kinetic modeling, we elucidate the pressure-dependent reaction pathways and identify key rate-limiting steps. A comprehensive mechanism is proposed, with emphasis on pressure-adaptive chemistry and intermediate species evolution. Model validation was achieved through comparison with steady-state ammonia measurements and optical emission data. Additionally, path flux and sensitivity analyses were conducted to deepen mechanistic insights and guide future process optimization under high-pressure conditions.

## Experiment and Modeling

### Experimental Setup

Figure 1 shows a schematic of the experimental setup used in this study. The experiments were conducted in a specially designed high-pressure coaxial DBD plasma reactor. A quartz tube with an inner diameter of 4 mm and a wall thickness of 1 mm was employed as the dielectric barrier. Prior to each experiment, the quartz surface was cleaned with ethanol to remove surface contaminants. The surface remained visually unchanged after repeated operation, with no detectable discoloration or roughening, indicating negligible aging effects during the study. A stainless-steel mesh (20 mm in length) was wrapped around the outer surface of the quartz tube as the ground electrode, while a stainless-steel rod (2 mm diameter) was inserted along the axis of the tube as the high-voltage electrode, creating a 1 mm discharge gap between the rod and the inner wall of the quartz tube. The flow rates of N_2_ and H_2_ were controlled by mass flow controllers (Bronkhorst, F-201CV), and the gases were thoroughly mixed at a fixed total flow rate of 40 mL/min with an N_2_/H_2_ ratio of 1:3 before entering the DBD reactor. This stoichiometric ratio was chosen based on thermodynamic equilibrium and plasma reaction kinetics considerations, ensuring sufficient hydrogen availability for nitrogen hydrogenation. The reactor pressure was controlled using a high-pressure regulator (Festo, MS2-LR), allowing adjustment in 1 bar increments within the range of 1–3 bar. Further details are provided in Section S1. The maximum operating pressure was limited to 3 bar due to both the mechanical constraints of the quartz reactor (wall thickness 1 mm) and the maximum voltage output of the alternating-current (AC) power supply. The gas temperature in the discharge region was measured using a fiber-optic thermometer (Omega, FOB102) equipped with a nonconductive probe that can be directly inserted into the discharge zone. This type of sensor is immune to electromagnetic interference and provides reliable temperature readings under high-voltage plasma conditions, thereby ensuring accurate monitoring of the local gas temperature during operation. The DBD was ignited by a high-voltage AC power supply (CORONA Lab, CTP-2000 K), operating at a peak-to-peak voltage of up to 30 kV and a frequency of 11 kHz. A high-voltage probe (Tektronix, P6015A) was employed to measure the applied voltage, while a current monitor (Bergoz, CT-E0.5) was used to record the current. All electrical signals were digitized and recorded using a four-channel digital oscilloscope (Tektronix, MDO 3024). It is worth noting that the accumulated charge in the DBD was quantified using the voltage across an external 0.47 μF capacitor connected between the ground electrode and ground. The discharge power was calculated based on the Q-U Lissajous figure.^29^

**Figure 1:**
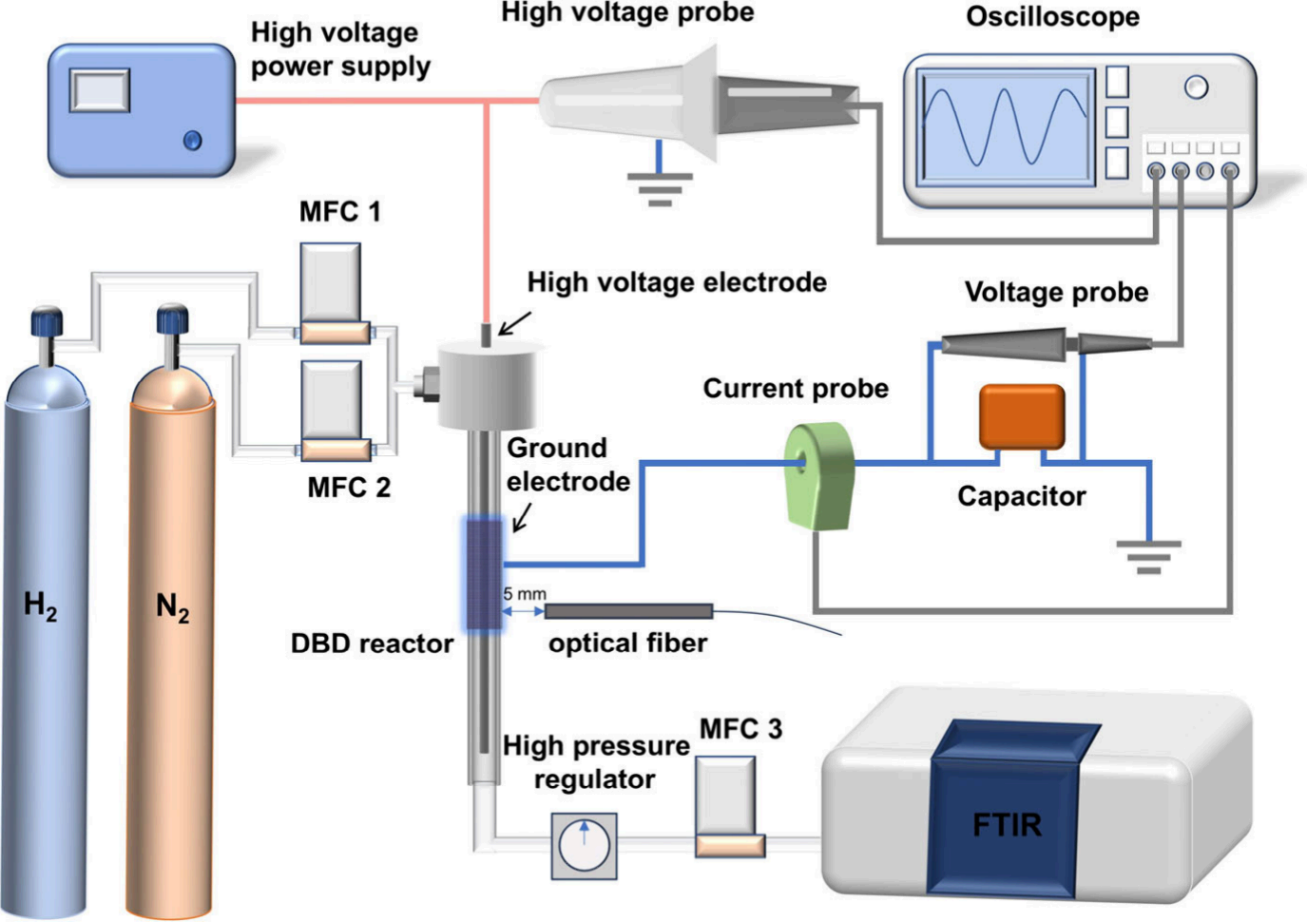
Schematic diagram of the experimental setup.

Quantitative analysis of gaseous products, including reactants (N_2_ and H_2_) and product (NH_3_), was conducted in real time using a Fourier transform infrared (FTIR) spectrometer (FTIR-4200, Jasco) with a resolution of 2 cm^–1^ (Figure S1). To ensure accuracy and reduce experimental error, each measurement was repeated three times. In addition, optical emission spectroscopy (OES) diagnostics of the N_2_/H_2_ DBD was carried out using an optical fiber connected to an Acton intensified charge-coupled device spectrometer (Model 320 PI, Princeton Instruments) equipped with a 600 grooves/mm grating. The optical fiber was positioned 5 mm from the ground electrode to collect light emitted from the discharge region. All measurements were conducted in a darkened environment to minimize interference from ambient light. Background spectra were recorded prior to plasma ignition and subsequently subtracted from the raw data to ensure accurate spectral analysis. Emission spectra were recorded over the wavelength range of 200–800 nm, with an exposure time of 0.5 s and a focal length of 320 mm.

The specific energy input (SEI) and energy yield (EY) are defined as^30,31^

1 $$\mathrm{SEI}\;\left(\mathrm{eV}/\mathrm{molecule}\right)=\frac{\mathrm{discharge power}\;\left(\mathrm{kW}\right)}{\mathrm{flow rate}\;\left(L/\min\right)}\times \frac{60\;s/\min\times 6.24\times 10^{21}\;\mathrm{eV}/\mathrm{kJ}\times V_{\mathrm{mol}}\;\left(L/\mathrm{mol}\right)}{6.02\times 10^{23}\;\mathrm{molecules}/\mathrm{mol}}$$

2 $$\mathrm{EY}\;\left(g/\mathrm{kWh}\right)=\frac{\mathrm{ammonia concentration}\;\left(\mathrm{ppm}\right)\times \mathrm{flow rate}\;\left(L/\min\right)\times 60\times 17}{\mathrm{discharge power}\;\left(\mathrm{kW}\right)\times 24.5\;L/\mathrm{mol}\times 1000000}$$

where *V*_mol_ is the molar volume.

### Kinetic Model

A spatially homogeneous plasma kinetics model was developed to investigate the underlying mechanism governing plasma-based ammonia synthesis under both ambient and elevated pressures. ZDPlasKin, a specialized plasma kinetics solver, was employed to simulate the temporal evolution of species densities by numerically integrating the continuity equation.^32^ The reduced electric field (E/N), a key parameter characterizing the plasma property, was iteratively determined at each time step based on the calculated power density.^33,34^

3 $$\frac{E}{N}=\frac{1}{N}\sqrt{\frac{p}{\sigma }}=\frac{1}{N}\sqrt{\frac{p}{en_{e}\mu_{e}}}$$

Here, *p* denotes the power density, *e* is the elementary charge (1.602176634 × 10^–19^ C), and σ, *n*_e_, and μ_e_ represent the electron conductivity, electron number density, and electron mobility, respectively, all determined using BOLSIG+.

In this study, we proposed a comprehensive chemistry set for plasma-based ammonia synthesis, incorporating electron-induced reactions, neutral–neutral interactions, ion–neutral reactions, electron–ion recombination reactions, and processes involving excited species. Table 1 lists all of the species considered in the model, including ground-state molecules, radicals, excited species, and ions. For electron-induced reactions, rate coefficients were computed using electron-impact cross sections via the Boltzmann equation solver BOLSIG+, which is integrated into ZDPlasKin.^35^ Most of the electron-impact cross sections for N_2_ and H_2_ were obtained from the Phelps database,^36^ while those for dissociative ionization and dissociative attachment of N_2_ and H_2_ were sourced from the Itikawa database.^37^ For NH_3_, cross sections for dissociative attachment, vibrational excitation, dissociation, and ionization were provided by the Morgan database,^38^ with additional dissociative ionization data also obtained from Itikawa.^39^ The gas-phase chemical reactions involving atoms, ground-state molecules, and radicals were primarily adopted from work by Gordiets et al.^40^ and Capitelli et al.^41^ It is important to note that the reaction NH_2_ + H (+M) → NH_3_ (+M) is pressure-dependent because its rate constant varies due to the involvement of third-body collisions.^42,43^ To accurately reflect high-pressure conditions, we implemented a Troe pressure falloff formulation for this reaction, as recommended in the literature.^43^

**Table 1:** Species Included in the Kinetic Model of Plasma-Assisted Ammonia Synthesis

species	symbol	number
molecules	N_2_, H_2_, NH_3_	3
atoms/radicals	H, N, NH, NH_2_	4
excited species	H_2_(v1), H_2_(v2), H_2_(v3), N_2_(v1), N_2_(v2), N_2_(v3), N_2_(v4), N_2_(v5), N_2_(v6), N_2_(v7), N_2_(v8), N_2_(A^3^), N_2_(B^3^), N_2_(a′^1^), N_2_(C^3^), N(^2^D), N(^2^P)	17
ions	H^+^, H_2_^+^, H_3_^+^, N^+^, N_2_^+^, N_3_^+^, N_4_^+^, NH^+^, NH_2_^+^, NH_3_^+^, NH_4_^+^, N_2_H^+^, H^–^, e	14

A detailed kinetic framework encompassing both vibrational and electronic excitation pathways was implemented within the plasma reaction mechanism. Energy exchange processes involving vibrational–translational (V–T) and vibrational–vibrational (V–V) interactions for excited molecular species were explicitly included. The rate coefficients for V–T and V–V processes were evaluated using the Schwartz–Slawsky–Herzfeld (SSH) theory.^41^ Additionally, the reverse reaction rate coefficients for V–V′ transition between N_2_ and H_2_ molecules were calculated using eq 4:^44^

4 $$Q_{nm}^{ls}=Q_{mn}^{sl}\;\exp\left(-\frac{\left(E_{s}^{B}-E_{l}^{B}\right)+\left(E_{m}^{A}-E_{n}^{A}\right)}{kT}\right)$$

where *Q*_*mn*_^*ls*^ denotes the probability of reverse V–V′ energy transfer between species A and B initially in vibrational states *m* and *s*, respectively, transitioning to final states *n* and *l*.

Furthermore, chain-branching reactions between vibrationally excited species and reactive radicals or atoms were recognized as key contributors to the effective utilization of vibrational energy. The rate constants for such reactions were calculated using the Fridman–Macheret α model,^45^ with computational details provided by Sun et al.^46^ To account for electronic kinetics, the model incorporated transitions among electronically excited nitrogen states [e.g., N_2_(A^3^) and N_2_(B^3^)] induced by vibrational excitation, optical transitions, and collisional interactions between electronically excited species and neutral particles. The relevant reaction rate coefficients were sourced from Capitelli et al.,^41^ Sun et al.,^47^ and Hong et al.^44^ Additionally, ion chemistry was comprehensively included in our model, covering ion–neutral reactions, ion–electron recombination, and mutual neutralization between cations and anions. The kinetic parameters were collected from the UMIST Database for Astrochemistry (https://udfa.ajmarkwick.net), as well as from Capitelli et al.^41^ and Hong et al.^44^

The relaxation of vibrational and electronic excitation due to surface interactions with the reactor wall was also considered. This wall-quenching effect was described using Chantry’s formula:^44^

5 $$k_{\mathrm{wall}}={\left(\tau_{\mathrm{wall}}\right)}^{-1}={\left[\frac{\Lambda^{2}}{D}+\frac{V}{A}\frac{2\left(2-\gamma_{\mathrm{wall}}\right)}{\mathrm{v̅}\gamma_{\mathrm{wall}}}\right]}^{-1}$$

where Λ denotes the diffusion length, *D* is the diffusion coefficient, ν is the thermal velocity of the species, and *V*/*A* is the volume-to-surface area ratio of the reactor. In this study, a *V*/*A* ratio of 0.075 cm was adopted, based on a discharge volume (*V*) of 0.1885 cm^3^ and a wall surface area (*A*) of 2.5133 cm^2^ within the discharge region. For a cylindrical reactor with a simple geometry, the diffusion length can be approximated by dividing the reactor radius (*R*) by 2.405. A diffusion coefficient of 7.9 × 10^–5^ m^2^/s at 300 K and 1 bar was assumed for both nitrogen and hydrogen species, with temperature and pressure dependencies accounted for using standard scaling laws. The wall loss probability γ_wall_ was assumed to be 1 × 10^–3^ for electronically excited N_2_*,^44,48^ 4.5 × 10^–4^ for N_2_(v), and 1.0 × 10^–4^ for H_2_(v).^44^ Ion losses at the wall were not considered for the following two reasons. First, ion mobility is strongly limited in highly collisional plasmas, leading to an ion velocity at the sheath edge that is significantly lower than the Bohm velocity. Second, the ion speed becomes particularly small due to the extremely short mean free path at atmospheric and elevated pressures.^48^ In addition, previous studies have demonstrated that wall-related ion losses are negligible.^49^ Overall, the kinetic mechanism for plasma-based NH_3_ synthesis in an N_2_/H_2_ mixture comprises 38 species (Table 1) and 500 elementary reactions (Tables S1–S10).

In experimental systems, AC-driven DBDs typically exhibit a filamentary discharge mode, characterized by the presence of microdischarge filaments with weak plasma between them.^50^ van’t Veer et al. proposed a method to systematically characterize the conditions of DBD by defining the power density as a time-resolved function.^50^ This approach enables the 0D model to account for the spatial and temporal nature of filamentary discharges through explicit consideration of the number of microdischarges, thereby scaling the model from filamentary to effectively uniform plasma conditions. To simulate this discharge character, a time-dependent, periodically pulsed power density profile was implemented by introducing a power density distribution factor, γ. Both the intense microdischarges and their postdischarge afterglows were incorporated into the model. The profile of the power density, including both filamentary and diffuse components, was calibrated based on measured electrical characteristics. This approach enabled a realistic estimation of radical densities and transient species compositions under practical plasma conditions. Further details on the temporal characterization of the power input are provided by van’t Veer et al.^50^ Furthermore, during the plasma discharge process, the actual gas temperature within the discharge filaments is higher than the bulk temperature, leading to a nonuniform temperature distribution. However, the volumetric fraction of these filaments is very small relative to the total discharge volume, and accurately determining the local filament temperature remains extremely challenging. Therefore, the bulk gas temperatures, measured using a fiber-optic probe, were directly employed as input parameters in the simulations. The key input parameters, including the temperature for each experimental condition, are provided in Table S11.

## Results and Discussion

### Validation of the Plasma Kinetic Model

To assess the validity of the developed plasma kinetic model, NH_3_ concentrations and energy yields obtained from both experiments and simulations were compared across a range of discharge powers and pressures. As shown in Figure 2a–c, the ammonia concentration exhibits a systematic increase with increasing discharge power, which is primarily attributed to enhanced energy deposited into the reacting gas mixture, facilitating more frequent and energetic electron–molecule collisions. However, elevated pressures were found to suppress ammonia formation in the plasma, contrary to the pressure-enhanced trend typically observed in conventional thermal catalytic HB process. At 1 bar, NH_3_ concentration increases from 790.8 to 1762.0 ppm with rising discharge power, while at 3 bar, a similar but lower increase from 563.8 to 1551.3 ppm is observed over the same power range. This behavior suggests that, although higher pressure thermodynamically favors NH_3_ formation by shifting the equilibrium, it simultaneously results in a reduction of the reduced electric field strength (E/N), as shown in Figure 4b. The weakened E/N significantly diminishes electron energy and thereby imposes kinetic limitations, such as reduced rates of electronic excitation and electron-impact dissociation, which are critical for ammonia synthesis under plasma conditions. The pronounced decreasing trend in SEI also indicates that the energy deposited on each molecule becomes insufficient to dissociate N_2_ molecules with increasing pressures. As a result, the thermodynamic gains from increased pressure are outweighed by kinetic losses, leading to overall lower ammonia concentrations at higher pressures. This trade-off highlights the importance of optimizing plasma operating parameters to balance kinetic and thermodynamic effects.

**Figure 2:**
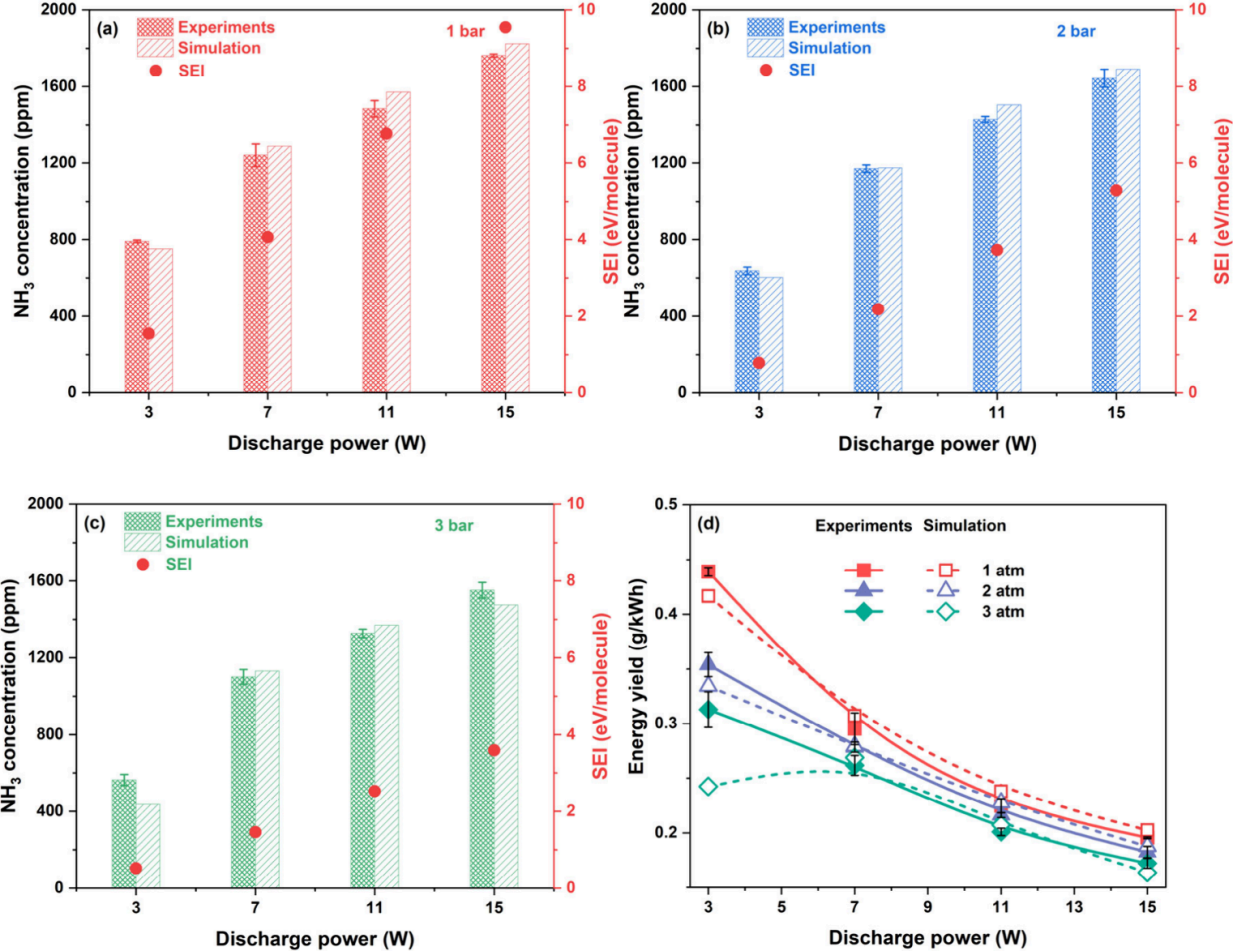
Comparison of NH_3_ concentrations and SEI between experimental measurements and model predictions as a function of discharge power at different pressures: (a) 1 bar; (b) 2 bar; (c) 3 bar. (d) Comparison of energy yield versus discharge power at different pressures.

Figure 2d illustrates the variation of NH_3_ energy yield as a function of discharge power. The energy yield decreases with increasing discharge power across all tested pressures. This decline is attributed to inefficient energy utilization at high discharge power, where an excess population of low-energy electrons fails to effectively participate in productive activation of N_2_ and H_2_ molecules. Instead, these electrons contribute to nonselective processes or dissipate energy through inelastic collisions, resulting in lower conversion and reduced energy yield.

Notably, the model predictions demonstrate excellent agreement with steady-state experimental measurements, with a relative deviation of less than 10%. While there are some discrepancies between the calculated and measured ammonia concentrations, these differences are not unexpected given the complexity of the reaction network and the considerable uncertainties associated with the rate coefficients of several reactions. Importantly, we deliberately avoid “tuning” the model solely to improve agreement with experimental data without a rigorous scientific justification. All assumptions in the model are based on logical and physically plausible principles.^33^ This strong correlation supports the fidelity and robustness of the developed kinetic mechanism, indicating that it effectively captures the essential physicochemical pathways governing plasma-based NH_3_ synthesis under varying operational conditions.

### OES Diagnostics

To understand the formation of reactive species in the N_2_/H_2_ DBD and elucidate the role of reactive species in plasma-assisted ammonia synthesis, we recorded the OES spectra at different pressures. Figure 3 shows a typical emission spectrum recorded for an N_2_/H_2_ DBD (N_2_/H_2_ ratio = 1:3) under 1 bar at a discharge power of 7 W. The most intense spectral features correspond to the N_2_ second positive system (SPS) (*C* ^3^Π_u_ → *B* ^3^Π_g_) in the range of 290.0–445.0 nm.^29^ A weaker band, corresponding to the N_2_ first positive system (FPS) (*B* ^3^Π_g_ → *A* ^3^∑_u_) is also detected in the range of 630.0–680.0 nm.^29^ The presence of these two N_2_ bands indicates electronic excitation of N_2_ by electron impact (e + N_2_ → e + N_2_*), a critical process in the activation of molecular nitrogen. Additionally, the N_2_^+^ first negative system (FNS) (*B* ^2^∑_u_^+^ → *X* ^2^∑_g_^+^) with a band head at 391.4 nm is clearly observed (Figure 3c), confirming ionization of N_2_ via e + N_2_ → 2e + N_2_^+^.^51,52^ It is worth noting that the emission bands of the N_2_^+^ FNS partially overlap with the N_2_ SPS, potentially complicating spectral deconvolution in this region.

**Figure 3:**
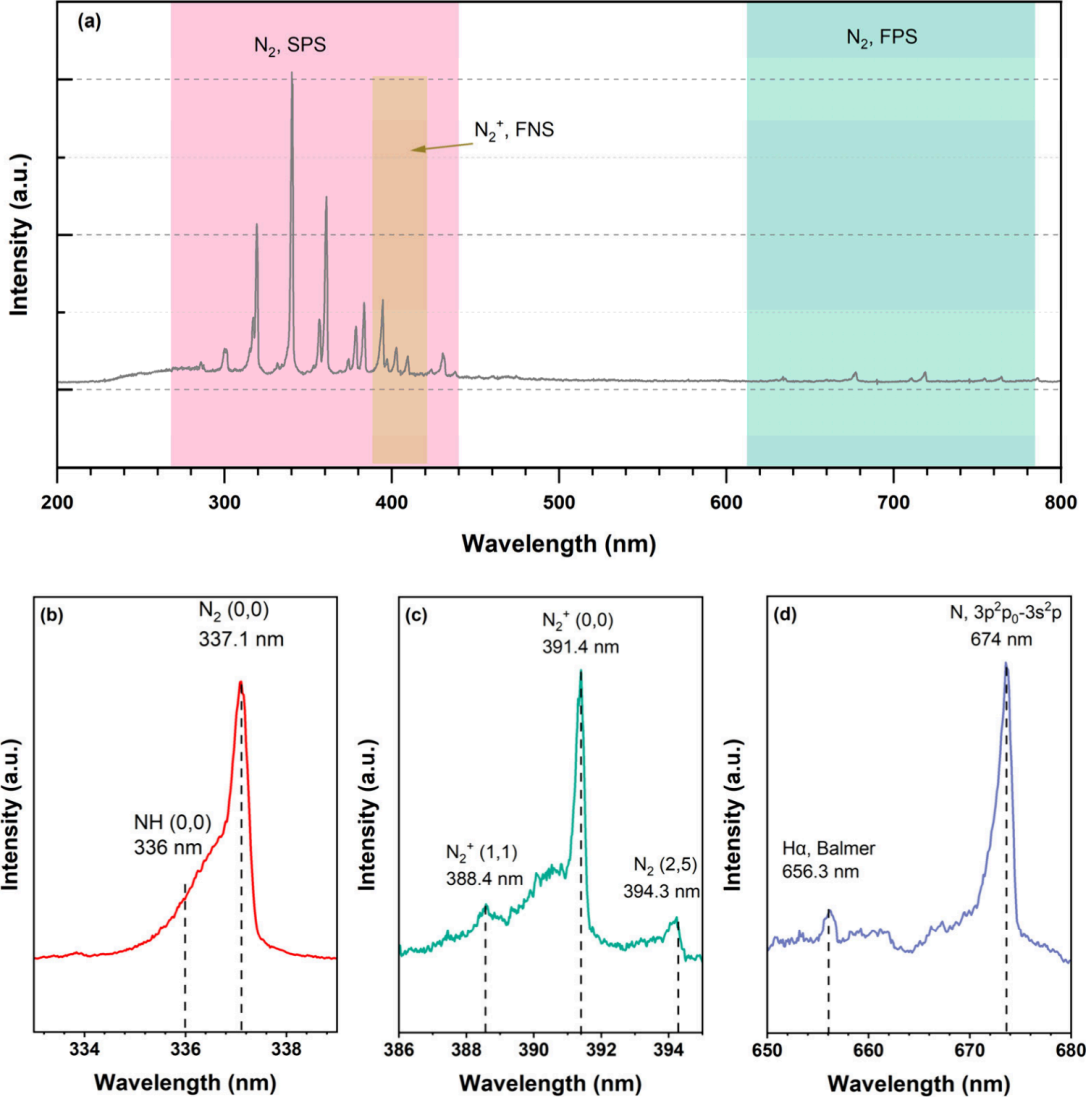
(a) Full-range emission spectra (200–800 nm), (b) NH band, (c) N_2_^+^ band, and (d) H and N atomic lines recorded for a N_2_/H_2_ DBD with a N_2_/H_2_ ratio of 1:3 at a discharge power of 7 W under 1 bar pressure.

NH radicals are identified through their *A* ^3^Π → *X* ^3^∑^–^ electronic transition, appearing as a shoulder peak at ∼360.0 nm, adjacent to the prominent 337.1 nm peak of the N_2_ SPS (*C* ^3^Π_u_ → *B* ^3^Π_g_, Δ*v* = 0). The presence of this shoulder feature indicates that NH_3_ dissociation (e + NH_3_ → e + NH + H_2_) and ionization (e + NH_3_ → 2e + NH + H_2_^+^) processes occur under the plasma conditions (Figure 3b).^29^ In the visible region of the spectrum, the most prominent hydrogen feature is the H_α_ line at 656.3 nm, attributed to the Balmer series transition (*n* = 3 → *n* = 2) of atomic hydrogen (Figure 3d).^53^ Furthermore, a distinct characteristic peak at 674.0 nm, attributed to the 3p^2^ P_0_ → 3s^2^ P transition of atomic nitrogen, is observed (Figure 3d). The simultaneous observation of both H_α_ and atomic N emission lines provides compelling evidence for the dissociation of H_2_ and N_2_ molecules into atomic species, a prerequisite for NH_3_ formation.

### Underlying Mechanism of Plasma-Driven Ammonia Synthesis at Elevated Pressure

The reduced electric field, E/N, plays a decisive role in electron energy transfer pathways and the formation of reactive species in plasma chemistry. Figure 4 illustrates the influence of E/N on the distribution of electron energy across various excitation and dissociation processes in an N_2_/H_2_ DBD (N_2_/H_2_ ratio = 1:3). At low E/N values (<10 Td), rotational excitation dominates, consuming most of the electron energy. As E/N increases into the range of 10–50 Td, the energy transfer becomes increasingly efficient toward vibrational excitation of N_2_, whereas the vibrational excitation efficiency of H_2_ gradually declines with increasing E/N.

**Figure 4:**
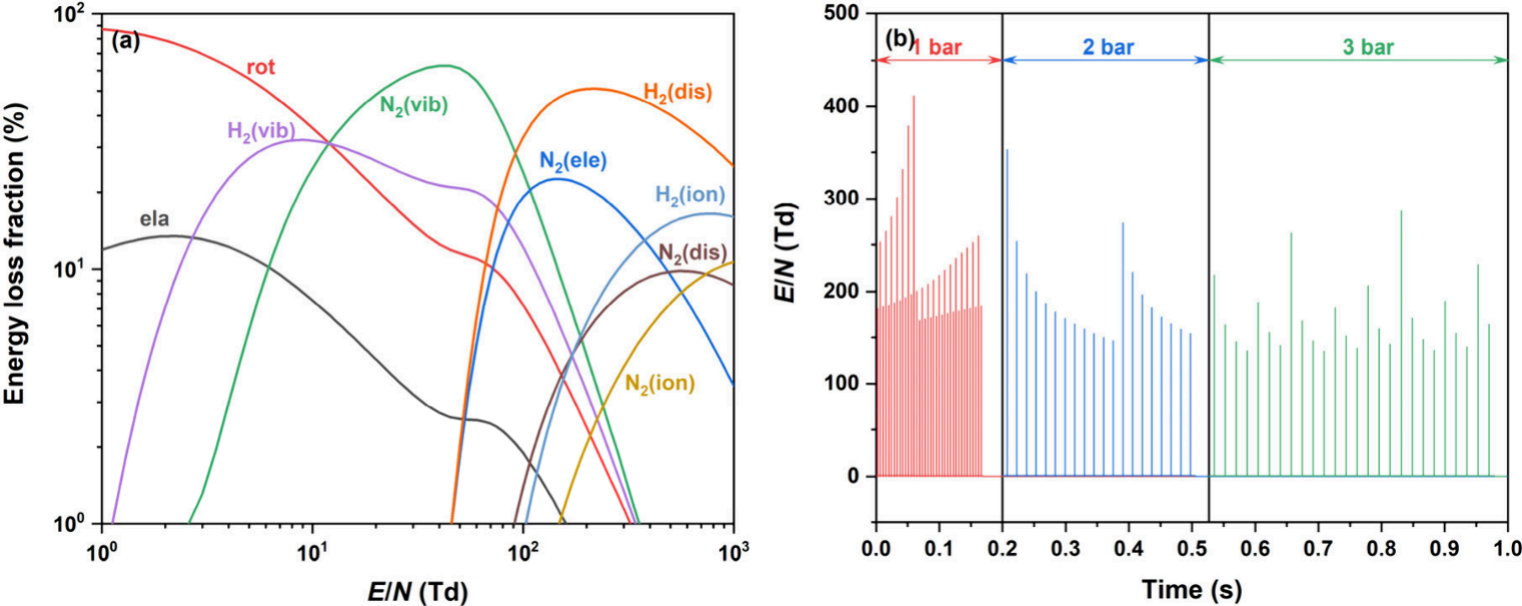
(a) Branching of electron energy dissipation into various excitation channels in a N_2_/H_2_ DBD with a N_2_/H_2_ ratio of 1:3 at varying E/N values (ela, elastic collision; rot, rotational; vib, vibrational excitation; ele, electronic excitation; dis, dissociation; ion, ionization). (b) Time-solved E/N at different pressures.

In our model, E/N fluctuates dynamically, reaching several hundred Td during microdischarges and dropping to a few Td during the afterglow phases between discharges. When averaged over time, E/N spans a range of 50–230 Td, within which the dominant energy dissipation channels shift toward H_2_ dissociation and N_2_ vibrational and electronic excitation. This dynamic behavior highlights the time-resolved complexity of plasma energy distribution under typical DBD conditions. As pressure increases, the total gas number density also rises, leading to a reduction in the average E/N, as shown in Figure 4b. As a result, the energy flux allocated to H_2_ dissociation weakens, while the electron energy directed toward N_2_ vibrational excitation correspondingly intensifies. This inverse relationship between pressure and E/N imposes kinetic limitations on key reaction pathways, particularly those requiring higher electron energies. At high E/N values (>230 Td), H_2_ dissociation becomes the primary electron energy sink, and the electric field becomes sufficiently strong to initiate gas ionization. However, despite the decreased E/N at elevated pressures, the Lissajous figures (Figure 5a) and the associated voltage and current waveforms (Figure 5c) remain remarkably consistent across all pressure conditions. This consistency suggests that the macroscopic discharge character, such as filament formation and plasma stability, remains fundamentally unaltered, even though the underlying microphysical energy partitioning is pressure-sensitive.

**Figure 5:**
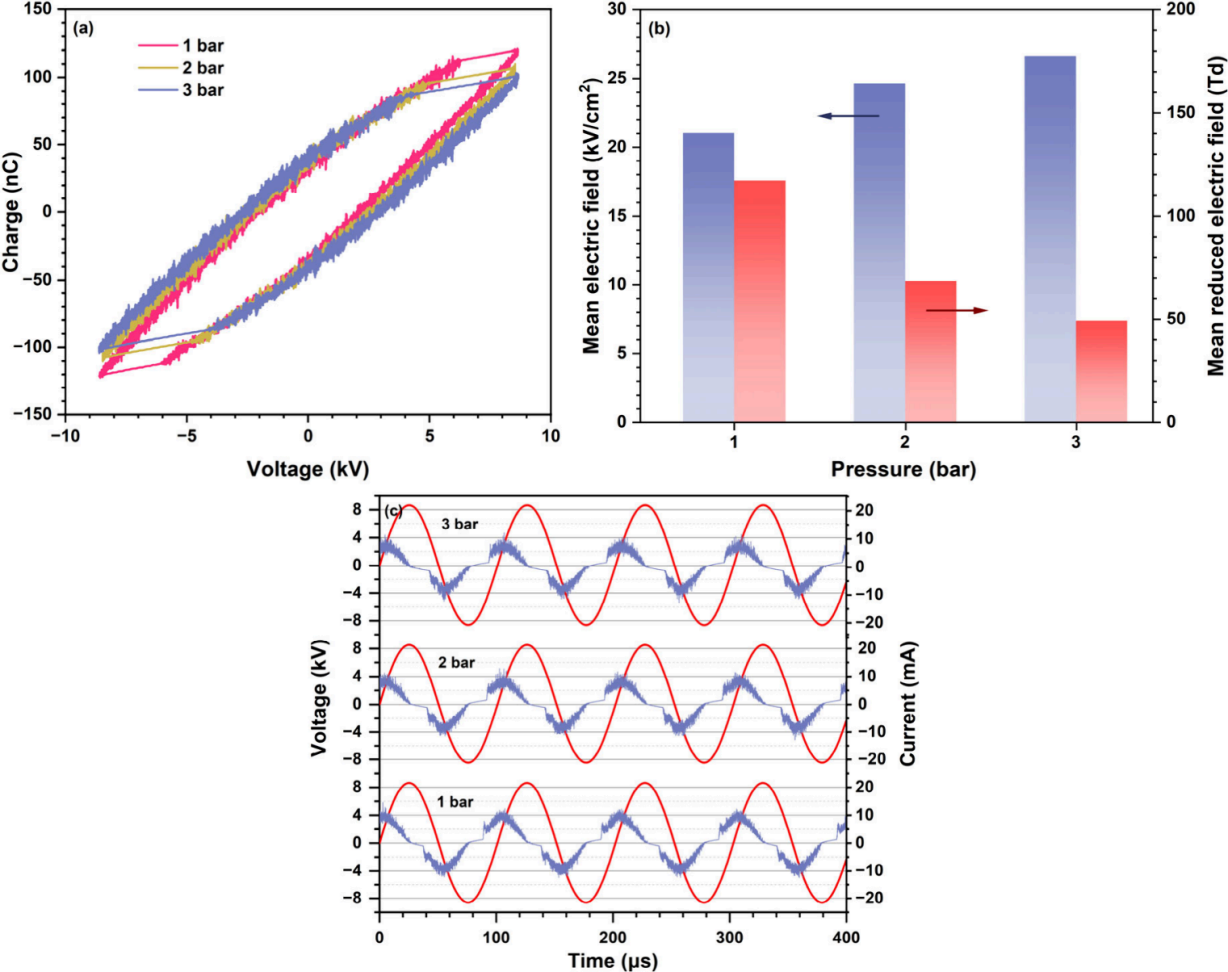
Comparison of (a) Lissajous figures, (b) mean electric field and reduced electric field under different pressures, and (c) voltage and current profiles of the N_2_/H_2_ DBD (N_2_/H_2_ ratio of 1:3) at a discharge power of 7 W and under different pressures.

To investigate the formation behavior of the main species in plasma-assisted ammonia synthesis under pressures ranging from 1 to 3 bar, we present the time-resolved evolution of the number densities of the most important species in Figure 6. It can be observed that these species exhibit a qualitatively similar temporal trend across all pressure conditions. In general, microdischarges, due to their nanosecond (ns) time scale, induce pulsed fluctuations in the concentrations of atoms, radicals, electronically excited species, and NH_3_, with pulse widths extending into the millisecond (ms) domain. During each microdischarge, electron density increases rapidly due to the ionization of both N_2_ and H_2_. Atomic N and its electronically excited N­(^2^D) are initially generated through electron-impact reactions. In addition to the direct dissociation of N_2_ by electrons, the dissociation of electronically excited N_2_(A^3^) significantly contributes to the formation of atomic N, resulting in a higher concentration of N compared to N­(^2^D). Subsequently, N and N­(^2^D) undergo successive hydrogenation, yielding NH and NH_2_ radicals. The concentration of NH_3_ gradually increases over time, primarily driven by radical recombination pathways, notably NH_2_ + H­(+M) → NH_3_(+M) and NH + H_2_ + M → NH_3_ + M. However, during the interdischarge phase between successive microdischarges, a decline in NH_3_ concentration is observed. This reduction is attributed to the dissociation of NH_3_, primarily facilitated by interactions with electronically excited species such as N_2_(A^3^) and N­(^2^D), as supported by the reaction flux analysis in Figure 7. Notably, this dissociation process coincides with a concurrent rise in NH_2_ concentration, indicating a dynamic equilibrium between ammonia synthesis and decomposition pathways that is strongly modulated by the pulsed nature of the discharge and the prevailing pressure conditions.

**Figure 6:**
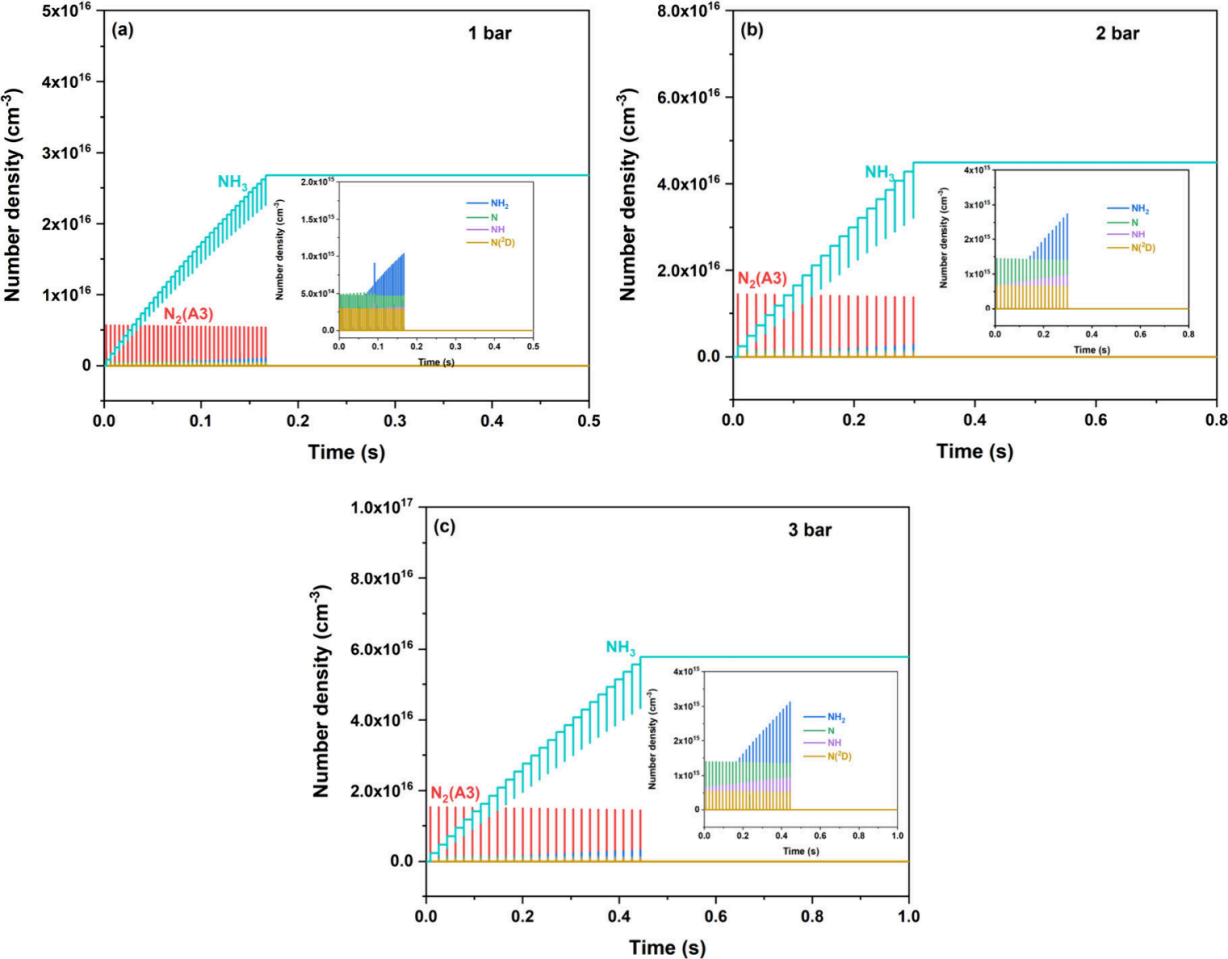
Time-dependent evolution of main species at a discharge power of 15 W under different pressures: (a) 1 bar; (b) 2 bar; (c) 3 bar.

**Figure 7:**
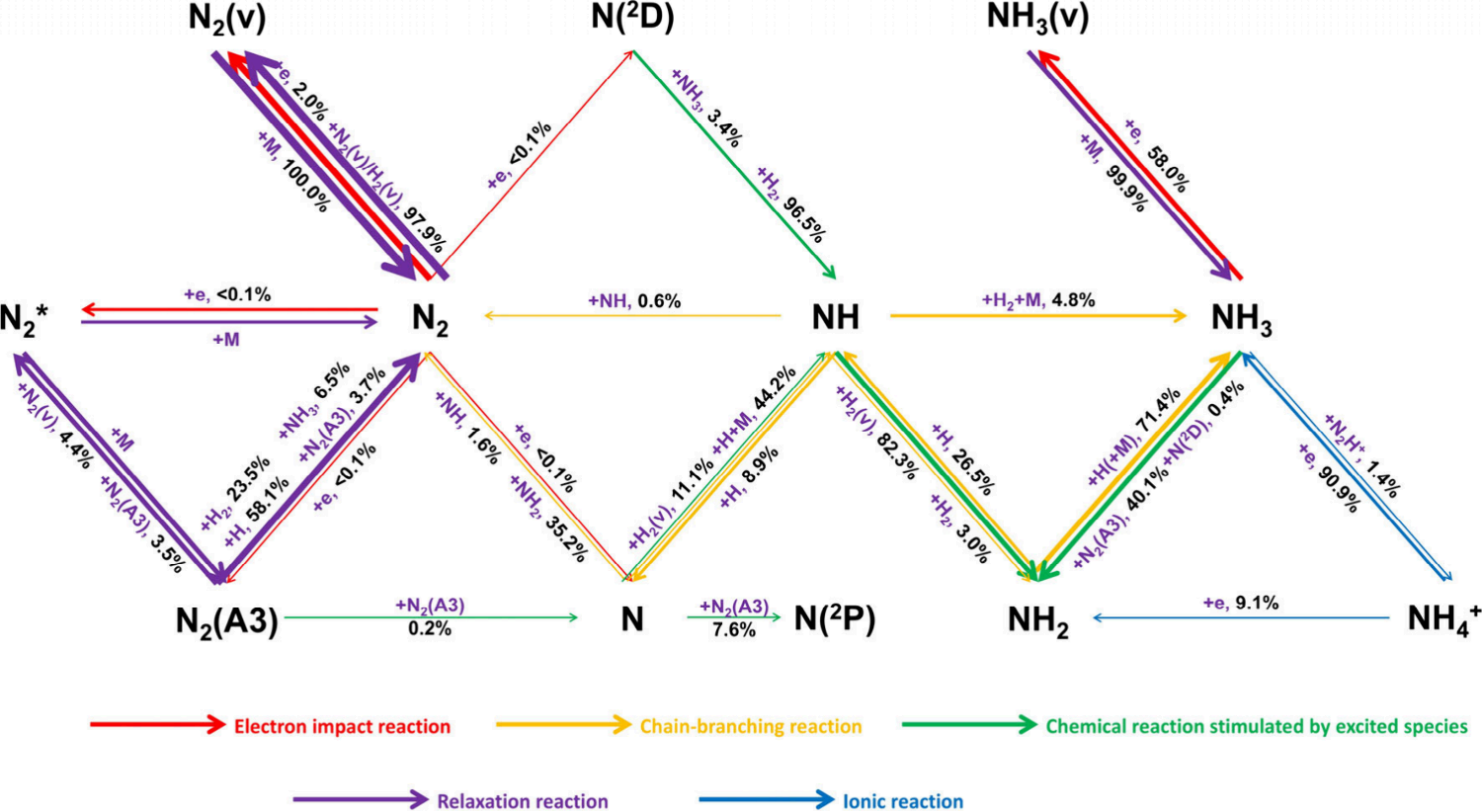
Network of plasma-assisted ammonia synthesis in a N_2_/H_2_ mixture (N_2_/H_2_ ratio 1:3) at 3 bar and 15 W discharge power [N_2_* includes N_2_(B^3^), N_2_(a′^1^), and N_2_(C^3^)].

To elucidate the underlying mechanism of ammonia formation under elevated pressure conditions, we present a quantitative reaction pathway flux analysis for plasma-assisted ammonia synthesis at a pressure of 3 bar and a discharge power of 15 W, as shown in Figure 7. For comparison, corresponding analyses were also conducted at 1 and 2 bar, with the results presented in Figure S3. The contributions to species formation or consumption were obtained by integrating the reaction rates over the entire residence time, thereby capturing the cumulative influence of each reaction pathway. The arrow thickness in the diagram encodes the magnitude of net production or consumption fluxes: the thickest arrows represent dominant reactions with contributions on the order of 10^–4^ mol/cm^3^, whereas the thinnest arrows correspond to minor pathways contributing around 10^–8^ mol/cm^3^. The numerical annotations attached to the arrows indicate the relative percentage contribution of each reaction to the formation or depletion of the relevant species, thereby providing a comprehensive visualization of the kinetic landscape under high-pressure conditions.

A significant portion of N_2_ is consumed through electron-impact vibrational excitation, e + N_2_ → e + N_2_(v), resulting in the formation of vibrationally excited N_2_(v). More than 99.9% of the N_2_(v) population is quenched back to the ground state N_2_ via V–T energy transfer, which contributes to thermalization, and a temperature rise in the N_2_/H_2_ mixture. Only a minor fraction of N_2_ undergoes dissociation via electron impact to produce N and N­(^2^D), or is excited electronically to form N_2_(A^3^). Specifically, at 3 bar, the model reveals that 0.2% of N_2_(A^3^) is consumed via the reaction N_2_(A^3^) + N_2_(A^3^) → N_2_ + N + N, representing the primary pathway for atomic N formation under these conditions. This result highlights the critical role of N_2_(A^3^) in promoting the NTP-assisted synthesis of ammonia from the N_2_/H_2_ mixture. Among the N atoms generated, 1.6% and 35.2% recombine with NH and NH_2_ radicals via N + NH → N_2_ + H and N + NH_2_ → N_2_ + H_2_/2H, respectively, thereby regenerating N_2_. Additionally, 7.6% of atomic N is excited by N_2_(A^3^) to form N­(^2^P) through the reaction N + N_2_(A^3^) → N­(^2^P) + N_2_. Beyond these two pathways, the three-body recombination reaction N + H + M → NH + M significantly contributes to N atom consumption, accounting for 44.2% of total N consumption. Furthermore, chain-branching reactions induced by excited N­(^2^D), particularly N­(^2^D) + H_2_ → NH + H and N­(^2^D) + NH_3_ → NH + NH_2_, are the key pathways for the production of NH radicals. It is noteworthy that the NH radicals are predominantly generated via the reactions N­(^2^D) + H_2_ → NH + H and NH_2_ + H → NH + H_2_ rather than through the direct recombination of N and H atoms, as suggested in ref 27. This finding is consistent at both 1 and 2 bar, as illustrated in Figure S3. Importantly, vibrationally excited hydrogen, H_2_(v), accelerates NH consumption via NH + H_2_(v) → NH_2_ + H, a reaction responsible for 82.3% of total NH consumption. Additionally, 4.8% of NH radicals are converted into NH_3_ through the recombination reaction NH + H_2_ + M → NH_3_ + M, which contributes 3.5% to total NH_3_ formation. NH_2_ radicals primarily combine with H atoms to form NH_3_, accounting for 71.4% of total NH_2_ consumption. From the perspective of NH_3_ formation, this pressure-dependent reaction NH_2_ + H­(+M) → NH_3_(+M) is the dominant pathway, contributing 90.8% of total NH_3_ production. This finding further supports the previously proposed pathway in which NH_3_ is formed via the hydrogenation of NH and NH_2_ radicals.^27^ In contrast, 26.5% of NH_2_ is consumed via dehydrogenation to NH. As shown in Figure 7, the consumption of NH_3_ via electron-impact dissociation and ionization is negligible due to the relatively low concentration of NH_3_. The primary NH_3_ decomposition pathway involves dissociation by N_2_(A^3^) and N­(^2^D) to form NH_2_ radicals. Electron-impact vibrational excitation [e + NH_3_ → e + NH_3_(v)] also contributes significantly, accounting for 58.0% of total NH_3_ consumption. However, this pathway can be effectively neglected, as more than 99% of NH_3_(v) relaxes back to the ground state via deexcitation reactions. Furthermore, the ionic reaction NH_3_ + N_2_H^+^ → NH_4_^+^ + N_2_ represents a minor pathway, contributing 1.4% to total NH_3_ loss. The resulting NH_4_^+^ ions primarily recombine with electrons to regenerate NH_3_, although 9.1% of NH_4_^+^ undergoes dissociative recombination to form NH_2_ radicals. Compared with the reaction pathways observed at 1 and 2 bar, the primary reaction pathways remain unchanged, indicating that increased pressure does not fundamentally alter the underlying kinetic structure of plasma-assisted ammonia synthesis.

To further identify rate-limiting steps, we performed a logarithmic sensitivity analysis, using the following definition for the sensitivity coefficient *lS*,^34^

6 $$lS=\frac{\log\left({\mathrm{Conc}}^{\prime }/\mathrm{Conc}\right)}{\log\left({k_{j}}^{\prime }/k_{j}\right)}$$

Here, *k*_*j*_′ and *k*_*j*_ are the rate coefficients for the *j*th reaction with and without doubling, respectively, while Conc′ and Conc represent the ammonia concentrations corresponding to these rate coefficients. A positive sensitivity coefficient indicates that the corresponding reaction promotes NH_3_ formation, while a negative coefficient implies an inhibitory effect.

Figure 8 shows that the electron-impact dissociation reaction e + N_2_ → e + N + N­(^2^D) has the most significant promoting effect on ammonia formation. As illustrated in Figure 7, this reaction is a key pathway for generating N and electronically excited N­(^2^D) species, which undergo successive hydrogenation to form NH and NH_2_ radicals. These intermediates primarily lead to NH_3_ formation via the reaction NH_2_ + H­(+M) → NH_3_(+M), which also exhibits a strong promoting effect according to the path flux analysis. Additionally, the dissociation of H_2_ and electronically excited nitrogen N_2_(A^3^) shows substantial positive sensitivity coefficients, as both reactions enhance the generation of H and N species, respectively. In contrast, the ionization reaction e + H_2_ → H_2_^+^ + 2e exhibits the strongest negative sensitivity because it competes with the electron-impact dissociation of N_2_ [e + N_2_ → e + N + N­(^2^D)] for electron energy, thereby inhibiting the formation of reactive N and N­(^2^D) species, as shown in Figure 4. Similarly, the ionization of N_2_ (e + N_2_ → 2e + N_2_^+^) follows a comparable inhibitory mechanism. As discussed earlier, the dissociation of NH_3_ by N_2_(A^3^) represents the dominant pathway for NH_3_ consumption, contributing to its negative sensitivity coefficient. Moreover, side reactions such as NH_2_ + N → N_2_ + H_2_, NH_2_ + N → N_2_ + 2H, and NH_2_ + H → NH + H_2_ directly compete with the NH_2_ hydrogenation [NH_2_ + H­(+M) → NH_3_(+M)], further limiting net NH_3_ formation.

**Figure 8:**
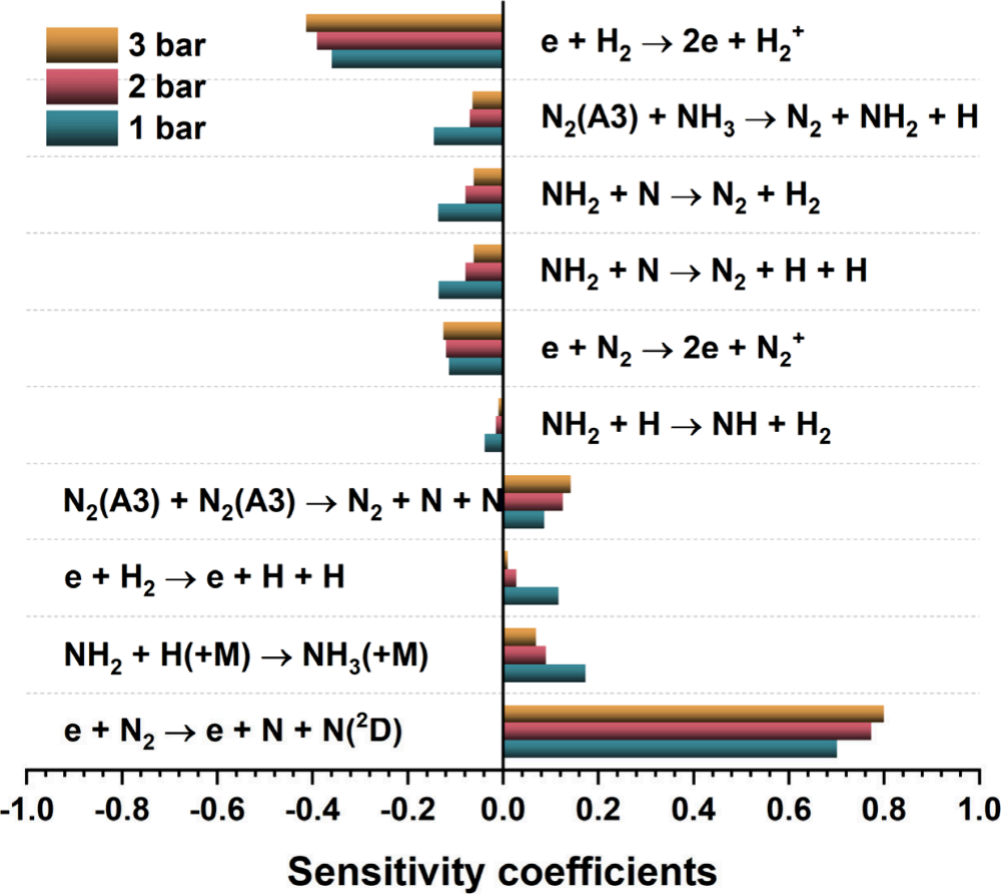
Sensitivity analysis for ammonia formation in plasma-assisted ammonia synthesis.

## Conclusions

In this work, we conducted a comprehensive experimental and chemical kinetic analysis to elucidate the underlying reaction mechanisms of plasma-driven ammonia synthesis at elevated pressure. The experimental results show a peak NH_3_ concentration of 1762.0 ppm at 15 W under 1 bar, compared to a lower NH_3_ concentration of 1551.3 ppm at the same discharge power under 3 bar, indicating that elevated pressure limits plasma ammonia synthesis. This effect is primarily due to the decrease in the reduced electric field at elevated pressure, which shifts the electron energy distribution toward lower-energy electrons and lowers the average electron energy. As a result, the formation of reactive nitrogen species such as N, N­(^2^D), and N_2_(A^3^) is limited, thereby suppressing the key kinetic pathways for ammonia synthesis. Consequently, the thermodynamic gains from increased pressure are outweighed by kinetic losses. Furthermore, the energy yield decreased with increasing discharge power at all tested pressures, primarily because the excess electrons generated at higher discharge power fail to effectively collide with target molecules (N_2_ and H_2_).

Plasma chemical kinetics modeling confirms that NTP-driven ammonia synthesis proceeds via the same reaction mechanism across a pressures range of 1–3 bar. The kinetic modeling results indicate that N_2_ is initially dissociated by electrons to produce atomic N and N­(^2^D) or excited through collisions to generate N_2_(A^3^). A small fraction of N_2_(A^3^) is consumed through the collision with N_2_(A^3^) to generate atomic N. N and N­(^2^D) are mainly hydrogenated via the three-body reaction N + H + M → NH + M and the reactions N + H_2_(v) → NH + H, and N­(^2^D) + H_2_ → NH + H, respectively. Vibrationally excited hydrogen, H_2_(v), consumes most of the NH radicals into NH_2_, while less than 5% of NH radicals proceed through the recombination with H_2_ to NH_3_. Finally, NH_2_ primarily combines with H atoms to form NH_3_ via the pressure-dependent reaction, which represents the dominant pathway for NH_3_ production. These results indicate that elevated pressure does not alter the fundamental mechanism of plasma-assisted ammonia synthesis. Sensitivity analysis conducted within the global kinetic model reveals that the electron-impact N_2_ dissociation reaction has the most significant promoting effect on ammonia formation, confirming it as the rate-limiting step. In contrast, the ionization reaction e + H_2_ → H_2_^+^ + 2e exhibits the highest negative sensitivity because it competes with the electron-impact dissociation of N_2_ for electron energy. The kinetic investigation of plasma-assisted ammonia synthesis at elevated pressure not only facilitates a deeper understanding of this complex reaction but also provides critical insights to inform reactor design and process optimization strategies. This work advances the fundamental understanding of plasma-assisted ammonia synthesis under elevated pressure, providing valuable mechanistic insights that support the optimization of plasma conditions and reactor design for improved efficiency and integration in future sustainable ammonia production technologies.

Despite the progress demonstrated in this study, several opportunities exist to further advance plasma-assisted ammonia synthesis. First, incorporating a suitable catalyst could lower the activation energy for N≡N triple-bond dissociation, thereby enhancing reaction kinetics and increasing ammonia yield. Second, the use of nanosecond-pulsed-discharge-driven DBD may generate intense electric fields on nanosecond time scales, efficiently promoting N_2_ dissociation while minimizing thermal effects, ultimately shifting the thermodynamic equilibrium toward ammonia formation. Finally, the limited availability of *in situ* diagnostics restricts experimental validation of intermediate species and mechanistic pathways. Future studies employing techniques such as laser-induced fluorescence and molecular-beam mass spectrometry could provide detailed insights into intermediates and confirm kinetic model predictions, supporting the optimization of plasma conditions and reactor design.

## Supplementary Material



## Notes

Details for pressure adjustment, qualitative analysis of NH_3_ by FTIR, overview of reactions included in the kinetic model, summary of input parameters used in the kinetic model, calculation of the mean reduced electric field, and reaction pathways for plasma-assisted ammonia synthesis at 1 and 2 bar (PDF)
