# Supplementary material for: Elevated Pressure Effects on Plasma-Driven Ammonia Synthesis: Insights from Experiments and Kinetic Modeling
Source: ACS Sustain Chem Eng. 2025 Sep 11;13(37):15576–87. doi: 10.1021/acssuschemeng.5c06251 (PMC12458982; doi:10.1021/acssuschemeng.5c06251)
Supplement: Supplementary file 1 [file sc5c06251_si_001.pdf]

# Supporting Information

# Elevated Pressure Effects on Plasma-Driven Ammonia Synthesis: Insights from Experiments and Kinetic Modeling

Jintao Sun[+], Weitao Wang[+], Chunqiang Lu, Xin Tu[*]

Department of Electrical Engineering and Electronics, University of Liverpool, Liverpool, L69 3GJ, UK

[+] J. Sun and W. Wang contributed equally to this work

* Corresponding author

E-mail address: xin.tu@liv.ac.uk (X. Tu)

**Number of pages**: 22

**Number of figures**: 3

**Number of tables**: 11

# Table of Contents

**S.1. Details for pressure adjustment** ........ S3

**S.2. Qualitative analysis of $NH_3$ by FTIR** ........ S4

**S.3. Overview of reactions included in the kinetic model** ........ S5

**S.4. Summary of input parameters used in the kinetic model** ........ S17

**S.5. Calculation of the mean reduced electric field** ........ S19

**S.6. Reaction pathways for plasma-assisted ammonia synthesis at 1 bar and 2 bar** ........ S20

**Reference** ........ S21

## S.1. Details for pressure adjustment

In our experimental setup, the pressure was precisely controlled using a high-pressure regulator in combination with an additional mass flow controller (MFC) positioned downstream. As illustrated in Figure 1 of the manuscript, both MFC1 and MFC2 are initially operated, while the flow rate of MFC 3 is kept at zero. Once the desired pressure in the reactor is reached, the flow rate of MFC3 is then adjusted to match the combined flow rates of MFC1 and MFC2.

## S.2. Qualitative analysis of $NH_3$ by FTIR

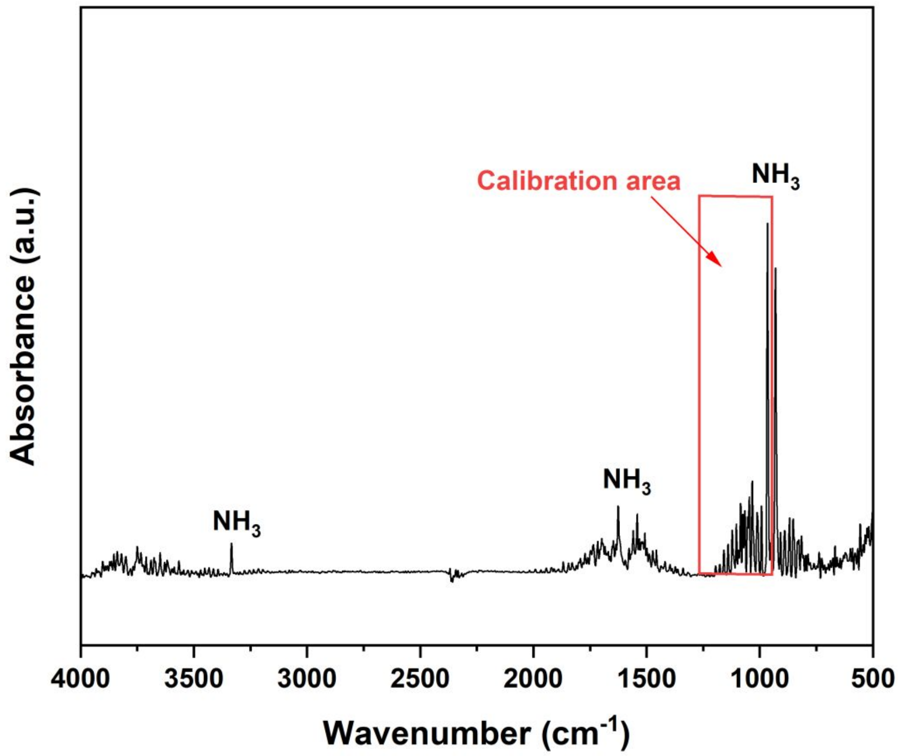


**Figure S1**. Typical FTIR spectrum of $NH_3$ obtained during plasma-driven ammonia synthesis.

## S.3. Overview of reactions included in the kinetic model

**Table S1.** Electron impact reactions

| No. | Reaction | Rate coefficients | Ref. |
|---|---|---|---|
| R1 | $e + H_2 \rightarrow H + H^-$ | f(σ,EEDF) | 1 |
| R2 | $e + NH_3 \rightarrow NH_2 + H^-$ | f(σ,EEDF) | 2 |
| R3 | $e + N_2 \rightarrow e + N_2(rot)$ | f(σ,EEDF) | 3 |
| R4a | $e + H_2 \rightarrow e + H_2(j0\text{-}2)$ | f(σ,EEDF) | 3 |
| R4b | $e + H_2 \rightarrow e + H_2(j1\text{-}3)$ | f(σ,EEDF) | 3 |
| R5a | $e + N_2 \rightarrow e + N_2(v1)$ | f(σ,EEDF) | 3 |
| R5b | $e + N_2 \rightarrow e + N_2(v1res)$ | f(σ,EEDF) | 3 |
| R6 | $e + N_2 \rightarrow e + N_2(v2)$ | f(σ,EEDF) | 3 |
| R7 | $e + N_2 \rightarrow e + N_2(v3)$ | f(σ,EEDF) | 3 |
| R8 | $e + N_2 \rightarrow e + N_2(v4)$ | f(σ,EEDF) | 3 |
| R9 | $e + N_2 \rightarrow e + N_2(v5)$ | f(σ,EEDF) | 3 |
| R10 | $e + N_2 \rightarrow e + N_2(v6)$ | f(σ,EEDF) | 3 |
| R11 | $e + N_2 \rightarrow e + N_2(v7)$ | f(σ,EEDF) | 3 |
| R12 | $e + N_2 \rightarrow e + N_2(v8)$ | f(σ,EEDF) | 3 |
| R13 | $e + H_2 \rightarrow e + H_2(v1)$ | f(σ,EEDF) | 3 |
| R14 | $e + H_2 \rightarrow e + H_2(v2)$ | f(σ,EEDF) | 3 |
| R15 | $e + H_2 \rightarrow e + H_2(v3)$ | f(σ,EEDF) | 3 |
| R16 | $e + NH_3 \rightarrow e + NH_3(v2)$ | f(σ,EEDF) | 2 |
| R17 | $e + NH_3 \rightarrow e + NH_3(v4)$ | f(σ,EEDF) | 2 |
| R18 | $e + NH_3 \rightarrow e + NH_3(v13)$ | f(σ,EEDF) | 2 |
| R19a | $e + N_2 \rightarrow e + N_2(A3, v0\text{-}4)$ | f(σ,EEDF) | 3 |
| R19b | $e + N_2 \rightarrow e + N_2(A3, v5\text{-}9)$ | f(σ,EEDF) | 3 |
| R19c | $e + N_2 \rightarrow e + N_2(A3, v10\text{-})$ | f(σ,EEDF) | 3 |
| R20a | $e + N_2 \rightarrow e + N_2(B3)$ | f(σ,EEDF) | 3 |
| R20b | $e + N_2 \rightarrow e + N_2(W3)$ | f(σ,EEDF) | 3 |
| R20c | $e + N_2 \rightarrow e + N_2(B'3)$ | f(σ,EEDF) | 3 |
| R21a | $e + N_2 \rightarrow e + N_2(a'1)$ | f(σ,EEDF) | 3 |

| | | | |
|---|---|---|---|
| R21b | e + $N_2$ → e + $N_2$(a1) | f(σ,EEDF) | 3 |
| R21c | e + $N_2$ → e + $N_2$(w1) | f(σ,EEDF) | 3 |
| R22a | e + $N_2$ → e + $N_2$(C3) | f(σ,EEDF) | 3 |
| R22b | e + $N_2$ → e + $N_2$(E3) | f(σ,EEDF) | 3 |
| R22c | e + $N_2$ → e + $N_2$(a″1) | f(σ,EEDF) | 3 |
| R23 | e + $N_2$ → e + N + N($^2$D) | f(σ,EEDF) | 3,4 |
| R24 | e + $H_2$ → e + H + H | f(σ,EEDF) | 3,5 |
| R25 | e + $NH_3$ → e + $NH_2$ + H | f(σ,EEDF) | 2 |
| R26 | e + $NH_3$ → e + NH + $H_2$ | f(σ,EEDF) | 2 |
| R27 | e + $N_2$→ e + e + $N_2^+$ | f(σ,EEDF) | 3 |
| R28 | e + $N_2$ → e + e + N + $N^+$ | f(σ,EEDF) | 1 |
| R29 | e + $H_2$ → e + e + $H_2^+$ | f(σ,EEDF) | 3 |
| R30 | e + $H_2$ → e + e + H + $H^+$ | f(σ,EEDF) | 1 |
| R31 | e + $NH_3$ → e + e + $NH_3^+$ | f(σ,EEDF) | 2 |
| R32 | e + $NH_3$ → e + e + H + $NH_2^+$ | f(σ,EEDF) | 6 |
| R33 | e + $NH_3$ → e + e + $H_2$ + $NH^+$ | f(σ,EEDF) | 6 |
| R34 | e + $NH_3$ → e + e + $NH_2$ + $H^+$ | f(σ,EEDF) | 6 |
| R35 | e + $NH_3$ → e + $H_2$ + H + $N^+$ | f(σ,EEDF) | 6 |
| R36 | e + $NH_3$ → e + e + NH + $H_2^+$ | f(σ,EEDF) | 6 |
| R37 | e + $H^-$ → e + e + H | f(σ,EEDF) | 7 |

**Table S2.** Vibrational relaxation reactions

| No. | Reaction | Rate coefficients | Ref. |
|---|---|---|---|
| R38 | $N_2$(v1) + $N_2$ → $N_2$ + $N_2$ | $7.80\times10^{-12}\times T_g\times\exp(-218.0/T_g^{1/3}+690.0/T_g)/(1-\exp(-0.29\times11605/T_g))$ | 8 |
| R39-R45 | $N_2$(vn) + $N_2$ → $N_2$(vn-1) + $N_2$ | SSH theory | 8 |
| R46 | $N_2$(v1) + $H_2$ → $N_2$ + $H_2$ | $4.90\times10^{-12}\times T_g\times\exp(-167.1/T_g^{1/3}+394.0/T_g^{2/3})/(1-\exp(-0.29\times11605/T_g))$ | 8 |
| R47-R53 | $N_2$(vn) + $H_2$ → $N_2$(vn-1) + $H_2$ | SSH theory | 8 |
| R54 | $N_2$(v1) + N → $N_2$ + N | $4.00\times10^{-10}\times(T_g/300.0)^{0.5}\times\exp(-7061.3/T_g)$ | 8 |
| R55-R61 | $N_2$(vn) + N → $N_2$(vn-1) + N | SSH theory | 8 |

| | | | |
|---|---|---|---|
| R62 | $N_2(v1) + H \rightarrow N_2 + H$ | $8.19\times10^{-10}\times\exp(-90.2/T_g^{1/3})\times\exp(-7146.6/T_g)$ | 9 |
| R63-R69 | $N_2(vn) + H \rightarrow N_2(vn\text{-}1) + H$ | SSH theory | 8 |
| R70 | $N_2(v1) + NH_3 \rightarrow N_2 + NH_3$ | $1.28\times10^{-07}\times\exp(-0.492\times3365.45^{0.302}\times T_g^{1/3})$ | 10 |
| R71-R77 | $N_2(vn) + NH_3 \rightarrow N_2(vn\text{-}1) + NH_3$ | SSH theory | 8 |
| R78-R133 | $N_2(vn) + N_2(vm) \rightarrow N_2(vn\text{-}1) + N_2(vm\text{+}1)$ | SSH theory | 8 |
| R134 | $H_2(v1) + H_2 \rightarrow H_2 + H_2$ | $5.83\times10^{-07}\times\exp(-227.0/T_g^{1/3}+529.0/T_g^{2/3})$ | 9 |
| R135-R136 | $H_2(vn) + H_2 \rightarrow H_2(vn\text{-}1) + H_2$ | SSH theory | 8 |
| R137 | $H_2(v1) + N_2 \rightarrow H_2 + N_2$ | $4.66\times10^{-08}\times\exp(-227.0/T_g^{1/3}+529.0/T_g^{2/3})$ | 9 |
| R138-R139 | $H_2(vn) + N_2 \rightarrow H_2(vn\text{-}1) + N_2$ | SSH theory | 8 |
| R140 | $H_2(v1) + NH_3 \rightarrow H_2 + NH_3$ | $0.23\times\exp(-19.94-144.9/T_g^{1/3})$ | 11 |
| R141-R142 | $H_2(vn) + NH_3 \rightarrow H_2(vn\text{-}1) + NH_3$ | SSH theory | 8 |
| R143 | $H_2(v1) + H \rightarrow H_2 + H$ | $1.73\times10^{-14}/T_g^{0.5}$ | 34 |
| R144-R145 | $H_2(vn) + H \rightarrow H_2(vn\text{-}1) + H$ | SSH theory | 8 |
| R146-R151 | $H_2(vn) + H_2(vm) \rightarrow H_2(vn\text{-}1) + H_2(vm\text{+}1)$ | SSH theory | 8 |
| R152 | $NH_3(v2) + N_2 \rightarrow NH_3 + N_2$ | $6.76\times10^{-17}\times8.25^{1/3}\times T_g^{0.5}\times$omega_NH₃N₂ [a] | 10,12 |
| R153 | $NH_3(v4) + N_2 \rightarrow NH_3 + N_2$ | $1.15\times10^{-15}\times8.25^{1/3}\times T_g^{0.5}\times$omega_NH₃N₂ [a] | 10,12 |
| R154 | $NH_3(v13) + N_2 \rightarrow NH_3 + N_2$ | $1.63\times10^{-20}\times8.25^{1/3}\times T_g^{0.5}\times$omega_NH₃N₂ [a] | 10,12 |
| R155 | $NH_3(v2) + H_2 \rightarrow NH_3 + H_2$ | $4.39\times10^{-17}\times1.79^{1/3}\times T_g^{0.5}\times$omega_NH₃H₂ [b] | 10,12 |
| R156 | $NH_3(v4) + H_2 \rightarrow NH_3 + H_2$ | $7.47\times10^{-16}\times1.79^{1/3}\times T_g^{0.5}\times$omega_NH₃H₂ [b] | 10,12 |
| R157 | $NH_3(v13) + H_2 \rightarrow NH_3 + H_2$ | $1.06\times10^{-20}\times1.79^{1/3}\times T_g^{0.5}\times$omega_NH₃H₂ [b] | 10,12 |
| R158 | $NH_3(v2) + NH_3 \rightarrow NH_3 + NH_3$ | $3.60\times10^{-11}$ | 13 |
| R159 | $NH_3(v4) + NH_3 \rightarrow NH_3 + NH_3$ | $3.60\times10^{-11}$ | 13 |
| R160 | $NH_3(v13) + NH_3 \rightarrow NH_3 + NH_3$ | $3.60\times10^{-11}$ | 13 |
| R161-R184 | $H_2(vn) + N_2(vm) \rightarrow H_2(vn\text{-}1) + N_2(vm\text{+}1)$ | SSH theory | 8,14 |
| R185-R208 | $H_2(vn) + N_2(vm) \rightarrow H_2(vn\text{+}1) + N_2(vm\text{-}1)$ | c | 14 |
| R209-R229 | $H_2(vn) + N_2(vm) \rightarrow H_2(vn\text{-}1) + N_2(vm\text{+}2)$ | SSH theory | 8,14 |
| R230-R250 | $H_2(vn) + N_2(vm) \rightarrow H_2(vn\text{+}1) + N_2(vm\text{-}2)$ | c | 14 |

a. omega_NH₃N₂=$1.155/(T_g/227.29)^{0.1462}+0.3945\times\exp(-0.6672\times(T_g/227.29))+2.05\times\exp(-2.168\times(T_g/227.29))$

b. omega_NH₃H₂=$1.155/(T_g/261.00)^{0.1462}+0.3945\times\exp(-0.6672\times(T_g/261.00))+2.05\times\exp(-2.168\times(T_g/261.00))$

c. calculated based on the equation (14) from literature [14].

**Table S3.** Neutral-neutral reactions

| No. | Reaction | Rate coefficients | Ref. |
|---|---|---|---|
| R251 | $N + N + N_2 \rightarrow N_2(A3) + N_2$ | $1.70\times10^{-33}$ | 15 |
| R252 | $N + N + H_2 \rightarrow N_2(A3) + H_2$ | $1.70\times10^{-33}$ | 15 |
| R253 | $N + N + N \rightarrow N_2(A3) + N$ | $1.00\times10^{-32}$ | 15 |
| R254 | $N + N + H \rightarrow N_2(A3) + H$ | $1.00\times10^{-32}$ | 15 |
| R255 | $N + N + N_2 \rightarrow N_2(B3) + N_2$ | $2.40\times10^{-33}$ | 15 |
| R256 | $N + N + H_2 \rightarrow N_2(B3) + H_2$ | $2.40\times10^{-33}$ | 15 |
| R257 | $N + N + N \rightarrow N_2(B3) + N$ | $1.40\times10^{-32}$ | 15 |
| R258 | $N + N + H \rightarrow N_2(B3) + H$ | $1.40\times10^{-32}$ | 15 |
| R259 | $N + N + N_2 \rightarrow N_2 + N_2$ | $8.30\times10^{-34}\times\exp(500.0/T_g)$ | 15 |
| R260 | $N + N + H_2 \rightarrow N_2 + H_2$ | $8.30\times10^{-34}\times\exp(500.0/T_g)$ | 15 |
| R261 | $H + H + H_2 \rightarrow H_2 + H_2$ | $8.80\times10^{-33}\times(300.0/T_g)^{0.60}$ | 15 |
| R262 | $H + H + N_2 \rightarrow H_2 + N_2$ | $8.30\times10^{-33}\times(300.0/T_g)$ | 15 |
| R263 | $NH + H + N_2 \rightarrow NH_2 + N_2$ | $1.00\times10^{-32}$ | 15 |
| R264 | $NH + H + H_2 \rightarrow NH_2 + H_2$ | $1.00\times10^{-32}$ | 15 |
| R265 | $NH + H_2 + N_2 \rightarrow NH_3 + N_2$ | $2.50\times10^{-35}\times(T_g/300.0)\times\exp(1700.0/T_g)$ | 15 |
| R266 | $NH + H_2 + H_2 \rightarrow NH_3 + H_2$ | $2.50\times10^{-35}\times(T_g/300.0)\times\exp(1700.0/T_g)$ | 15 |
| R267 | $N + H_2 \rightarrow NH + H$ | $3.90\times10^{-10}\times\exp(-15775.0/T_g)$ | 8 |
| R268 | $NH + H \rightarrow N + H_2$ | $8.30\times10^{-11}\times\exp(-1000.0/T_g)$ | 8 |
| R269 | $NH + N \rightarrow N_2 + H$ | $1.80\times10^{-11}\times(T_g/300.0)^{0.5}$ | 8 |
| R270 | $NH + H_2 \rightarrow NH_2 + H$ | $2.20\times10^{-11}\times\exp(-3880.0/T_g)$ | 8 |
| R271 | $NH + NH \rightarrow NH_2 + N$ | $5.80\times10^{-12}\times(T_g/300.0)^{0.5}\times\exp(-1000.0/T_g)$ | 8 |
| R272 | $NH + M \rightarrow N + H + M$ | $5.80\times10^{-08}/(T_g/300.0)^{2.0}\times\exp(-42000.0/T_g)$ | 8 |
| R273 | $NH_2 + H \rightarrow NH + H_2$ | $3.20\times10^{-11}$ | 8 |
| R274 | $NH_2 + N \rightarrow NH + NH$ | $4.00\times10^{-11}\times(T_g/300.0)^{0.5}\times\exp(-11890.0/T_g)$ | 8 |
| R275 | $NH_2 + H_2 \rightarrow NH_3 + H$ | $2.10\times10^{-11}\times(T_g/300.0)^{0.5}\times\exp(-9274.0/T_g)$ | 8 |
| R276 | $NH_2 + NH_2 \rightarrow NH_3 + NH$ | $1.00\times10^{-11}\times\exp(-5000.0/T_g)$ | 8 |
| R277 | $NH_2 + M \rightarrow NH + H + M$ | $5.80\times10^{-06}/(T_g/300.0)^{2.0}\times\exp(-46000.0/T_g)$ | 8 |

| | | | |
|---|---|---|---|
| R278 | $NH_3 + H \rightarrow NH_2 + H_2$ | $2.10\times10^{-10}\times\exp(-10820.0/T_g)$ | 8 |
| R279 | $NH_3 + NH \rightarrow NH_2 + NH_2$ | $7.40\times10^{-11}\times\exp(-15470.0/T_g)$ | 8 |
| R280 | $NH_3 + M \rightarrow NH + H_2 + M$ | $1.00\times10^{-09}\times\exp(-47000.0/T_g)$ | 8 |
| R281 | $NH_3 + M \rightarrow NH_2 + H + M$ | $4.20\times10^{-08}\times\exp(-47200.0/T_g)$ | 8 |
| R282 | $N_2 + H \rightarrow NH + N$ | $4.70\times10^{-10}\times(T_g/300.0)^{0.5}\times\exp(-75900.0/T_g)$ | 8 |
| R283 | $NH + NH \rightarrow N_2 + H_2$ | $5.00\times10^{-14}\times(T_g/300.0)$ | 15 |
| R284 | $NH + NH \rightarrow N_2 + H + H$ | $8.50\times10^{-11}$ | 15 |
| R285 | $NH_2 + N \rightarrow N_2 + H_2$ | $1.20\times10^{-10}$ | 15 |
| R286 | $NH_2 + N \rightarrow N_2 + H + H$ | $1.20\times10^{-10}$ | 15 |
| R287 | $NH_2 + NH \rightarrow NH_3 + N$ | $1.66\times10^{-12}$ | 15 |
| R288 | $N + H + M \rightarrow NH + M$ | $5.02\times10^{-32}$ | 27 |
| R289 | $N + H_2 + M \rightarrow NH_2 + M$ | $1.00\times10^{-36}$ | 27 |
| R290 | $NH_2 + H(+M) \rightarrow NH_3(+M)$ | High /$2.56\times10^{-10}\times T_g^{0.167}$/ [d]<br>Low /$7.17\times10^{-25}/T_g^{2.083}$/<br>Troe /0.5 $1.0\times10^{-30}$ $1.0\times10^{30}$ $1.0\times10^{30}$/ | 16 |

d: Troe pressure fall-off formulation

**Table S4.** Chain-branching reactions stimulated by vibrationally excited molecules

| No. | Reaction | Rate coefficients | Ref. |
|---|---|---|---|
| R291 | $H_2(v1) + N \rightarrow NH + H$ | $3.90\times10^{-10}\times\exp(-10882.4/T_g)$ | 17 |
| R292 | $H_2(v2) + N \rightarrow NH + H$ | $3.90\times10^{-10}\times\exp(-6293.2/T_g)$ | 17 |
| R293 | $H_2(v3) + N \rightarrow NH + H$ | $3.90\times10^{-10}\times\exp(-1552.3/T_g)$ | 17 |
| R294 | $H_2(v1) + NH \rightarrow NH_2 + H$ | $2.20\times10^{-11}$ | 17 |
| R295 | $H_2(v2) + NH \rightarrow NH_2 + H$ | $2.20\times10^{-11}$ | 17 |
| R296 | $H_2(v3) + NH \rightarrow NH_2 + H$ | $2.20\times10^{-11}$ | 17 |
| R297 | $H_2(v1) + NH_2 \rightarrow NH_3 + H$ | $2.10\times10^{-11}\times(T_g/300.0)^{0.5}\times\exp(-6597.5/T_g)$ | 17 |
| R298 | $H_2(v2) + NH_2 \rightarrow NH_3 + H$ | $2.10\times10^{-11}\times(T_g/300.0)^{0.5}\times\exp(-4087.1/T_g)$ | 17 |
| R299 | $H_2(v3) + NH_2 \rightarrow NH_3 + H$ | $2.10\times10^{-11}\times(T_g/300.0)^{0.5}\times\exp(-1493.6/T_g)$ | 17 |
| R300 | $N_2(v1) + H \rightarrow NH + N$ | $4.70\times10^{-10}\times(T_g/300.0)^{0.5}\times\exp(-72635.7/T_g)$ | 17 |
| R301 | $N_2(v2) + H \rightarrow NH + N$ | $4.70\times10^{-10}\times(T_g/300.0)^{0.5}\times\exp(-69258.7/T_g)$ | 17 |
| R302 | $N_2(v3) + H \rightarrow NH + N$ | $4.70\times10^{-10}\times(T_g/300.0)^{0.5}\times\exp(-65994.4/T_g)$ | 17 |

| | | | |
|---|---|---|---|
| R303 | $N_2(v4) + H \rightarrow NH + N$ | $4.70\times10^{-10}\times(T_g/300.0)^{0.5}\times\exp(-62730.0/T_g)$ | 17 |
| R304 | $N_2(v5) + H \rightarrow NH + N$ | $4.70\times10^{-10}\times(T_g/300.0)^{0.5}\times\exp(-59353.1/T_g)$ | 17 |
| R305 | $N_2(v6) + H \rightarrow NH + N$ | $4.70\times10^{-10}\times(T_g/300.0)^{0.5}\times\exp(-56088.7/T_g)$ | 17 |
| R306 | $N_2(v7) + H \rightarrow NH + N$ | $4.70\times10^{-10}\times(T_g/300.0)^{0.5}\times\exp(-52711.8/T_g)$ | 17 |
| R307 | $N_2(v8) + H \rightarrow NH + N$ | $4.70\times10^{-10}\times(T_g/300.0)^{0.5}\times\exp(-49447.4/T_g)$ | 17 |
| R308 | $NH_3(v2) + H \rightarrow NH_2 + H_2$ | $2.10\times10^{-10}\times\exp(-10057.4/T_g)$ | 17 |
| R309 | $NH_3(v4) + H \rightarrow NH_2 + H_2$ | $2.10\times10^{-10}\times\exp(-9514.6/T_g)$ | 17 |
| R310 | $NH_3(v13) + H \rightarrow NH_2 + H_2$ | $2.10\times10^{-10}\times\exp(-8105.7/T_g)$ | 17 |
| R311 | $NH_3(v2) + N \rightarrow NH_2 + NH$ | $4.52\times10^{-10}/T_g^{0.226}\times\exp(-13075.3/T_g)$ | 17 |
| R312 | $NH_3(v4) + N \rightarrow NH_2 + NH$ | $4.52\times10^{-10}/T_g^{0.226}\times\exp(-12100.6/T_g)$ | 17 |
| R313 | $NH_3(v13) + N \rightarrow NH_2 + NH$ | $4.52\times10^{-10}/T_g^{0.226}\times\exp(-9570.8/T_g)$ | 17 |
| R314 | $NH_3(v2) + NH \rightarrow NH_2 + NH_2$ | $7.40\times10^{-11}\times\exp(-14528.4/T_g)$ | 17 |
| R315 | $NH_3(v4) + NH \rightarrow NH_2 + NH_2$ | $7.40\times10^{-11}\times\exp(-13858.1/T_g)$ | 17 |
| R316 | $NH_3(v13) + NH \rightarrow NH_2 + NH_2$ | $7.40\times10^{-11}\times\exp(-12118.5/T_g)$ | 17 |

**Table S5.** Wall relaxation reactions

| No. | Reaction | Rate coefficients | Ref. |
|---|---|---|---|
| R317 | $N_2(A3)$ + wall $\rightarrow N_2$ + wall | $1.0\times10^{-03}$ [e] | 14 |
| R318 | $N_2(a'1)$ + wall $\rightarrow N_2(B3)$ + wall | $1.0\times10^{-03}$ [e] | 14 |
| R319 | $N_2(vi)$ + wall $\rightarrow N_2(vi-1)$ + wall | $4.5\times10^{-04}$ [e] | 14 |
| R320 | $H_2(vj)$ + wall $\rightarrow H_2(vj-1)$ + wall | $1.0\times10^{-04}$ [e] | 14 |

e: wall loss probability

**Table S6.** Reactions involving electronically excited species

| No. | Reaction | Rate coefficients | Ref. |
|---|---|---|---|
| R321 | $N_2(B3) \rightarrow N_2(A3)$ | $1.34\times10^{5}$ | 8 |
| R322 | $N_2(a'1) \rightarrow N_2$ | $1.00\times10^{2}$ | 8 |
| R323 | $N_2(C3) \rightarrow N_2(B3)$ | $2.45\times10^{7}$ | 8 |
| R324 | $N_2(A3) \rightarrow N_2$ | $5.00\times10^{-01}$ | 8 |
| R325 | $N(^2P) \rightarrow N(^2D)$ | $1.58\times10^{-01}$ | 18 |

| | | | |
|---|---|---|---|
| R326 | $N(^2D) \rightarrow N$ | $2.04\times10^{-05}$ | 19 |
| R327 | $N(^2P) \rightarrow N$ | $9.00\times10^{-02}$ | 19 |
| R328 | $N_2(A3) + N_2(v6) \rightarrow N_2(B3) + N_2$ | $3.00\times10^{-11}$ | 8,14 |
| R329 | $N_2(A3) + N_2(v7) \rightarrow N_2(B3) + N_2(v1)$ | $3.00\times10^{-11}$ | 8,14 |
| R330 | $N_2(A3) + N_2(v8) \rightarrow N_2(B3) + N_2(v2)$ | $3.00\times10^{-11}$ | 8,14 |
| R331 | $N_2(B3) + N_2 \rightarrow N_2(A3) + N_2(v6)$ | $3.00\times10^{-11}$ | 8,14 |
| R332 | $N_2(B3) + N_2(v1) \rightarrow N_2(A3) + N_2(v7)$ | $3.00\times10^{-11}$ | 8,14 |
| R333 | $N_2(B3) + N_2(v2) \rightarrow N_2(A3) + N_2(v8)$ | $3.00\times10^{-11}$ | 8,14 |
| R334 | $N_2(A3) + N \rightarrow N_2 + N$ | $2.00\times10^{-12}$ | 8 |
| R335 | $N_2(A3) + N \rightarrow N_2 + N(^2P)$ | $4.00\times10^{-11}\times(300.0/T_g)^{2/3}$ | 8 |
| R336 | $N_2(A3) + N_2 \rightarrow N_2 + N_2$ | $3.00\times10^{-16}$ | 8 |
| R337 | $N_2(A3) + H \rightarrow N_2 + H$ | $2.10\times10^{-10}$ | 8 |
| R338 | $N_2(A3) + H_2 \rightarrow N_2 + H + H$ | $2.00\times10^{-10}\times\exp(-3500.0/T_g)$ | 8 |
| R339 | $N_2(A3) + N_2(A3) \rightarrow N_2 + N_2(B3)$ | $3.00\times10^{-10}$ | 8 |
| R340 | $N_2(A3) + N_2(A3) \rightarrow N_2 + N_2(C3)$ | $1.50\times10^{-10}$ | 8 |
| R341 | $N_2(A3) + N_2(A3) \rightarrow N_2 + N + N$ | $3.00\times10^{-11}$ | 14 |
| R342 | $N_2(A3) + H \rightarrow NH + N$ | $1.41\times10^{-09}/T_g^{0.09}\times\exp(-73558.2/T_g)$ | 20 |
| R343 | $N_2(A3) + NH_3 \rightarrow N_2 + NH_2 + H$ | $1.50\times10^{-10}$ | 21 |
| R344 | $N_2(B3) + N_2 \rightarrow N_2 + N_2$ | $2.00\times10^{-12}$ | 8 |
| R345 | $N_2(B3) + H_2 \rightarrow N_2(A3) + H_2$ | $2.50\times10^{-11}$ | 8 |
| R346 | $N_2(B3) + N_2 \rightarrow N_2(A3) + N_2$ | $3.00\times10^{-11}$ | 14 |
| R347 | $N_2(B3) + N \rightarrow N_2(a'1) + N$ | $3.30\times10^{-11}$ | 22 |
| R348 | $N_2(B3) + N \rightarrow N_2(A3) + N$ | $1.00\times10^{-10}$ | 22 |
| R349 | $N_2(B3) + N_2 \rightarrow N_2(C3) + N_2$ | $3.30\times10^{-11}\times\exp(-42700.0/T_g)$ | 22 |
| R350 | $N_2(B3) + N_2(A3) \rightarrow N_2(C3) + N_2$ | $4.60\times10^{-10}$ | 22 |
| R351 | $N_2(B3) + H \rightarrow NH + N$ | $1.41\times10^{-09}/T_g^{0.09}\times\exp(-73558.2/T_g)$ | 23 |
| R352 | $N_2(a'1) + N_2 \rightarrow N_2(B3) + N_2$ | $1.90\times10^{-13}$ | 8 |
| R353 | $N_2(a'1) + H \rightarrow N_2 + H$ | $1.50\times10^{-10}$ | 8 |
| R354 | $N_2(a'1) + H_2 \rightarrow N_2 + H + H$ | $2.60\times10^{-11}$ | 8 |
| R355 | $N_2(a'1) + N_2(a'1) \rightarrow N_4^+ + e$ | $1.00\times10^{-11}$ | 8 |

| | | | |
|---|---|---|---|
| R356 | $N_2(a'1) + N_2(A3) \rightarrow N_4^+ + e$ | $4.00\times10^{-12}$ | 8 |
| R357 | $N_2(a'1) + N_2(a'1) \rightarrow N_2^+ + N_2 + e$ | $1.00\times10^{-11}$ | 14 |
| R358 | $N_2(a'1) + N_2 \rightarrow N_2(A3) + N_2$ | $2.00\times10^{-13}$ | 22 |
| R359 | $N_2(a'1) + N_2 \rightarrow N_2 + N_2$ | $6.00\times10^{-14}$ | 22 |
| R360 | $N_2(a'1) + N_2 \rightarrow N_2(C3) + N_2$ | $1.00\times10^{-11}\times\exp(-30500.0/T_g)$ | 22 |
| R361 | $N_2(a'1) + H \rightarrow NH + N$ | $1.41\times10^{-09}/T_g^{0.09}\times\exp(-73558.2/T_g)$ | 23 |
| R362 | $N_2(C3) + N_2 \rightarrow N_2(a'1) + N_2$ | $1.00\times10^{-11}$ | 8 |
| R363 | $N_2(C3) + N \rightarrow N_2(B3) + N$ | $3.30\times10^{-11}$ | 3 |
| R364 | $N_2(C3) + N_2 \rightarrow N_2(B3) + N_2(A3)$ | $4.60\times10^{-10}\times\exp(-28900.0/T_g)$ | 22 |
| R365 | $N_2(C3) + N_2 \rightarrow N_2 + N$ | $5.00\times10^{-11}$ | 22 |
| R366 | $N_2(C3) + N_2 \rightarrow N_2(B3) + N_2$ | $3.30\times10^{-11}$ | 22 |
| R367 | $N_2(C3) + H \rightarrow NH + N$ | $1.41\times10^{-09}/T_g^{0.09}\times\exp(-73558.2/T_g)$ | 23 |
| R368 | $N_2(C3) + H_2 \rightarrow N_2 + H + H$ | $3.00\times10^{-10}$ | 21 |
| R369 | $N(^2D) + N_2 \rightarrow N + N_2$ | $6.50\times10^{-15}$ | 8 |
| R370 | $N(^2D) + H_2 \rightarrow NH + H$ | $2.30\times10^{-12}$ | 8 |
| R371 | $N(^2D) + NH_3 \rightarrow NH + NH_2$ | $1.10\times10^{-10}$ | 8 |
| R372 | $N(^2D) + N \rightarrow N(^2P) + N$ | $1.04\times10^{-12}\times\exp(-13800.0/T_g)$ | 22 |
| R373 | $N(^2P) + N \rightarrow N(^2D) + N$ | $1.80\times10^{-12}$ | 8 |
| R374 | $N(^2P) + N_2 \rightarrow N + N_2$ | $2.00\times10^{-18}$ | 8 |
| R375 | $N(^2P) + H_2 \rightarrow H + NH$ | $2.50\times10^{-14}$ | 8 |
| R376 | $N(^2P) + N \rightarrow N + N$ | $1.80\times10^{-12}$ | 14 |
| R377 | $N(^2P) + H_2 \rightarrow N + H_2$ | $2.00\times10^{-15}$ | 24 |

**Table S7.** Positive ion-neutral reactions

| No. | Reaction | Rate coefficients | Ref. |
|---|---|---|---|
| R378 | $H^+ + H_2 + M \rightarrow H_3^+ + M$ | $3.10\times10^{-29}$ | 8 |
| R379 | $H^+ + NH \rightarrow NH^+ + H$ | $2.10\times10^{-09}\times(300.0/T_g)^{0.5}$ | 25 |
| R380 | $H^+ + NH_2 \rightarrow NH_2^+ + H$ | $2.90\times10^{-09}\times(300.0/T_g)^{0.5}$ | 25 |
| R381 | $H^+ + NH_3 \rightarrow NH_3^+ + H$ | $3.70\times10^{-09}\times(300.0/T_g)^{0.5}$ | 25 |
| R382 | $H^+ + H + M \rightarrow H_2^+ + M$ | $1.0\times10^{-34}$ | 26 |

| | | | |
|---|---|---|---|
| R383 | $H^+ + H_2 \rightarrow H_2^+ + H$ | $1.0\times10^{-10}\times\exp(-21200.0/T_g)$ | 22 |
| R384 | $H_2^+ + H_2 \rightarrow H_3^+ + H$ | $2.10\times10^{-09}$ | 8 |
| R385 | $H_2^+ + N_2 \rightarrow N_2H^+ + H$ | $1.95\times10^{-09}$ | 8 |
| R386 | $H_2^+ + H \rightarrow H^+ + H_2$ | $6.40\times10^{-10}$ | 25 |
| R387 | $H_2^+ + N \rightarrow NH^+ + H$ | $1.90\times10^{-09}$ | 25 |
| R388 | $H_2^+ + NH \rightarrow NH^+ + H_2$ | $7.60\times10^{-10}\times(300.0/T_g)^{0.5}$ | 25 |
| R389 | $H_2^+ + NH \rightarrow NH_2^+ + H$ | $7.60\times10^{-10}\times(300.0/T_g)^{0.5}$ | 25 |
| R390 | $H_2^+ + NH_2 \rightarrow NH_2^+ + H_2$ | $2.10\times10^{-09}\times(300.0/T_g)^{0.5}$ | 25 |
| R391 | $H_2^+ + NH_3 \rightarrow NH_3^+ + H_2$ | $5.70\times10^{-09}\times(300.0/T_g)^{0.5}$ | 25 |
| R392 | $H_2^+ + N_2 \rightarrow N_2^+ + H_2$ | $1.18\times10^{-11}\times\exp(-2000.0/T_g)$ | 22 |
| R393 | $H_3^+ + N_2 \rightarrow N_2H^+ + H_2$ | $1.80\times10^{-09}$ | 8 |
| R394 | $H_3^+ + NH_3 \rightarrow NH_4^+ + H_2$ | $5.00\times10^{-09}$ | 8 |
| R395 | $H_3^+ + NH \rightarrow NH_2^+ + H_2$ | $1.30\times10^{-09}\times(300.0/T_g)^{0.5}$ | 25 |
| R396 | $H_3^+ + NH_2 \rightarrow NH_3^+ + H_2$ | $1.80\times10^{-09}\times(300.0/T_g)^{0.5}$ | 25 |
| R397 | $N^+ + H_2 \rightarrow NH^+ + H$ | $6.00\times10^{-10}\times\exp(-150.0/T_g)$ | 8 |
| R398 | $N^+ + NH_3 \rightarrow NH_3^+ + N$ | $2.00\times10^{-09}$ | 8 |
| R399 | $N^+ + NH_3 \rightarrow N_2H^+ + H_2$ | $2.20\times10^{-10}$ | 8 |
| R400 | $N^+ + N + N_2 \rightarrow N_2^+ + N_2$ | $1.00\times10^{-29}$ | 8 |
| R401 | $N^+ + N_2 + N_2 \rightarrow N_3^+ + N_2$ | $1.70\times10^{-29}\times(300.0/T_g)^{2.1}$ | 8 |
| R402 | $N^+ + NH \rightarrow N_2^+ + H$ | $3.70\times10^{-10}\times(300.0/T_g)^{0.5}$ | 25 |
| R403 | $N^+ + NH \rightarrow NH^+ + N$ | $3.70\times10^{-10}\times(300.0/T_g)^{0.5}$ | 25 |
| R404 | $N^+ + NH_2 \rightarrow NH_2^+ + N$ | $1.00\times10^{-09}\times(300.0/T_g)^{0.5}$ | 25 |
| R405 | $N^+ + NH_3 \rightarrow NH_2^+ + NH$ | $2.16\times10^{-10}\times(300.0/T_g)^{0.5}$ | 25 |
| R406 | $N^+ + N_2 \rightarrow N_2^+ + N$ | $1.11\times10^{-11}\times\exp(-12600.0/T_g)$ | 22 |
| R407 | $N_2^+ + N \rightarrow N^+ + N_2$ | $7.20\times10^{-13}\times(T_g/300.0)$ | 8 |
| R408 | $N_2^+ + H_2 \rightarrow N_2H^+ + H$ | $1.70\times10^{-09}$ | 8 |
| R409 | $N_2^+ + NH_3 \rightarrow NH_3^+ + N_2$ | $1.90\times10^{-09}$ | 8 |
| R410 | $N_2^+ + N_2 + N \rightarrow N_3^+ + N_2$ | $9.00\times10^{-30}\times\exp(400.0/T_g)$ | 8 |
| R411 | $N_2^+ + N_2 + N_2 \rightarrow N_4^+ + N_2$ | $5.20\times10^{-29}\times(300.0/T_g)^{2.2}$ | 8 |
| R412 | $N_2^+ + N_2(A3) \rightarrow N_3^+ + N$ | $3.00\times10^{-10}$ | 20 |

| | | | |
|---|---|---|---|
| R413 | $N_2^+ + NH \rightarrow NH^+ + N_2$ | $6.50\times10^{-10}\times(300.0/T_g)^{0.5}$ | 25 |
| R414 | $N_2^+ + NH_2 \rightarrow NH_2^+ + N_2$ | $8.90\times10^{-10}\times(300.0/T_g)^{0.5}$ | 25 |
| R415 | $N_2^+ + H \rightarrow H^+ + N_2$ | $1.20\times10^{-10}$ | 22 |
| R416 | $N_2^+ + H_2 \rightarrow H_2^+ + N_2$ | $2.00\times10^{-11}$ | 22 |
| R417 | $N_3^+ + N \rightarrow N_2^+ + N_2$ | $6.60\times10^{-11}$ | 8 |
| R418 | $N_3^+ + H_2 \rightarrow N_2H^+ + NH$ | $2.00\times10^{-13}$ | 8 |
| R419 | $N_3^+ + NH_3 \rightarrow NH_3^+ + N_2 + N$ | $2.10\times10^{-09}$ | 8 |
| R420 | $N_3^+ + H_2 \rightarrow H_2^+ + N_2 + N$ | $1.10\times10^{-08}$ | 22 |
| R421 | $N_4^+ + N \rightarrow N^+ + N_2 + N_2$ | $1.00\times10^{-11}$ | 8 |
| R422 | $N_4^+ + N_2 \rightarrow N_2^+ + N_2 + N_2$ | $2.10\times10^{-16}\times\exp(T_g/121.0)$ | 8 |
| R423 | $N_4^+ + H_2 \rightarrow H_2^+ + N_2 + N_2$ | $3.00\times10^{-10}\times\exp(-1800.0/T_g)$ | 8 |
| R424 | $N_4^+ + H_2 \rightarrow N_2H^+ + N_2 + H$ | $1.07\times10^{-09}$ | 8 |
| R425 | $N_4^+ + NH_3 \rightarrow NH_3^+ + N_2 + N_2$ | $1.80\times10^{-09}$ | 8 |
| R426 | $N_4^+ + M \rightarrow N_2^+ + N_2 + M$ | $1.00\times10^{-10}\times\exp(-5000.0/T_g)$ | 22 |
| R427 | $NH^+ + H_2 \rightarrow NH_2^+ + H$ | $1.30\times10^{-09}$ | 8 |
| R428 | $NH^+ + H_2 \rightarrow H_3^+ + N$ | $2.30\times10^{-10}$ | 8 |
| R429 | $NH^+ + N_2 \rightarrow N_2H^+ + N$ | $6.50\times10^{-10}$ | 8 |
| R430 | $NH^+ + NH_3 \rightarrow NH_3^+ + NH$ | $1.80\times10^{-09}$ | 8 |
| R431 | $NH^+ + NH_3 \rightarrow NH_4^+ + N$ | $6.00\times10^{-10}$ | 8 |
| R432 | $NH^+ + N \rightarrow N_2^+ + H$ | $1.39\times10^{-09}$ | 25 |
| R433 | $NH^+ + NH \rightarrow NH_2^+ + N$ | $1.00\times10^{-09}\times(300.0/T_g)^{0.5}$ | 25 |
| R434 | $NH^+ + NH_2 \rightarrow NH_3^+ + N$ | $1.50\times10^{-09}\times(300.0/T_g)^{0.5}$ | 25 |
| R435 | $NH_2^+ + H_2 \rightarrow NH_3^+ + H$ | $1.60\times10^{-09}$ | 8 |
| R436 | $NH_2^+ + NH_3 \rightarrow NH_3^+ + NH_2$ | $6.90\times10^{-10}$ | 8 |
| R437 | $NH_2^+ + NH_3 \rightarrow NH_4^+ + NH$ | $1.60\times10^{-09}$ | 8 |
| R438 | $NH_2^+ + N \rightarrow N_2H^+ + H$ | $9.10\times10^{-11}$ | 25 |
| R439 | $NH_2^+ + NH \rightarrow NH_3^+ + N$ | $7.30\times10^{-10}\times(300.0/T_g)^{0.5}$ | 25 |
| R440 | $NH_2^+ + NH_2 \rightarrow NH_3^+ + NH$ | $1.00\times10^{-09}\times(300.0/T_g)^{0.5}$ | 25 |
| R441 | $NH_3^+ + H_2 \rightarrow NH_4^+ + H$ | $1.50\times10^{-09}\times\exp(-2570.0/T_g)$ | 8 |
| R442 | $NH_3^+ + NH_3 \rightarrow NH_4^+ + NH_2$ | $2.20\times10^{-09}$ | 8 |

| | | | |
|---|---|---|---|
| R443 | $NH_3^+ + NH \rightarrow NH_4^+ + N$ | $7.10\times10^{-10}\times(300.0/T_g)^{0.5}$ | 25 |
| R444 | $NH_3^+ + NH_2 \rightarrow NH_4^+ + NH$ | $1.00\times10^{-11}\times(300.0/T_g)^{0.5}$ | 25 |
| R445 | $N_2H^+ + NH \rightarrow NH_2^+ + N_2$ | $6.40\times10^{-10}\times(300.0/T_g)^{0.5}$ | 25 |
| R446 | $N_2H^+ + NH_2 \rightarrow NH_3^+ + N_2$ | $8.90\times10^{-10}\times(300.0/T_g)^{0.5}$ | 25 |
| R447 | $N_2H^+ + NH_3 \rightarrow NH_4^+ + N_2$ | $2.30\times10^{-09}\times(300.0/T_g)^{0.5}$ | 25 |

**Table S8.** Negative ion-neutral reactions

| No. | Reaction | Rate coefficients | Ref. |
|---|---|---|---|
| R448 | $H^- + H \rightarrow H_2 + e$ | $1.80\times10^{-09}$ | 8 |
| R449 | $H^- + N \rightarrow NH + e$ | $1.00\times10^{-09}$ | 25 |
| R450 | $H^- + NH \rightarrow NH_2 + e$ | $1.00\times10^{-10}$ | 25 |
| R451 | $H^- + NH_2 \rightarrow NH_3 + e$ | $1.00\times10^{-09}$ | 25 |
| R452 | $H^- + M \rightarrow H + e + M$ | $2.70\times10^{-10}\times(T_g/300.0)^{0.50}\times\exp(-5590.0/T_g)$ | 25 |

**Table S9.** Mutual neutralization reactions

| No. | Reaction | Rate coefficients | Ref. |
|---|---|---|---|
| R453 | $H^- + H_2^+ \rightarrow H + H + H$ | $2.00\times10^{-07}\times(300.0/T_g)$ | 14 |
| R454 | $H^- + H_3^+ \rightarrow H_2 + H + H$ | $2.00\times10^{-07}\times(300.0/T_g)$ | 14 |
| R455 | $H^- + N_2^+ \rightarrow N_2 + H$ | $2.00\times10^{-07}\times(300.0/T_g)$ | 14 |
| R456 | $H^- + N_4^+ \rightarrow N_2 + N_2 + H$ | $2.00\times10^{-07}\times(300.0/T_g)$ | 14 |
| R457 | $H^- + N_2H^+ \rightarrow N_2 + H_2$ | $2.00\times10^{-07}\times(300.0/T_g)$ | 14 |
| R458 | $H^- + H_2^+ + N_2 \rightarrow H_2 + H + N_2$ | $2.00\times10^{-25}\times(300.0/T_g)^{2.50}$ | 15 |
| R459 | $H^- + H_2^+ + H_2 \rightarrow H_2 + H + H_2$ | $2.00\times10^{-25}\times(300.0/T_g)^{2.50}$ | 15 |
| R460 | $H^- + H_3^+ + N_2 \rightarrow H_2 + H_2 + N_2$ | $2.00\times10^{-25}\times(300.0/T_g)^{2.50}$ | 15 |
| R461 | $H^- + H_3^+ + H_2 \rightarrow H_2 + H_2 + H_2$ | $2.00\times10^{-25}\times(300.0/T_g)^{2.50}$ | 15 |
| R462 | $H^- + N_2^+ + N_2 \rightarrow N_2 + H + N_2$ | $2.00\times10^{-25}\times(300.0/T_g)^{2.50}$ | 15 |
| R463 | $H^- + N_2^+ + H_2 \rightarrow N_2 + H + H_2$ | $2.00\times10^{-25}\times(300.0/T_g)^{2.50}$ | 15 |
| R464 | $H^- + N_4^+ + N_2 \rightarrow N_2 + N_2 + H + N_2$ | $2.00\times10^{-25}\times(300.0/T_g)^{2.50}$ | 15 |
| R465 | $H^- + N_4^+ + H_2 \rightarrow N_2 + N_2 + H + H_2$ | $2.00\times10^{-25}\times(300.0/T_g)^{2.50}$ | 15 |
| R466 | $H^- + N_2H^+ + N_2 \rightarrow N_2 + H_2 + N_2$ | $2.00\times10^{-25}\times(300.0/T_g)^{2.50}$ | 15 |

| | | | |
|---|---|---|---|
| R467 | $H^- + N_2H^+ + H_2 \rightarrow N_2 + H_2 + H_2$ | $2.00\times10^{-25}\times(300.0/T_g)^{2.50}$ | 15 |
| R468 | $H^- + H^+ \rightarrow H + H$ | $7.51\times10^{-08}\times(300.0/T_g)^{0.50}$ | 25 |
| R469 | $H^- + N^+ \rightarrow H + N$ | $7.51\times10^{-08}\times(300.0/T_g)^{0.50}$ | 6 |
| R470 | $H^- + NH_3^+ \rightarrow NH_3 + H$ | $7.51\times10^{-08}\times(300.0/T_g)^{0.50}$ | 25 |
| R471 | $H^- + NH_4^+ \rightarrow NH_3 + H + H$ | $7.51\times10^{-08}\times(300.0/T_g)^{0.50}$ | 25 |
| R472 | $H^- + N_2H^+ \rightarrow N_2 + H + H$ | $7.51\times10^{-08}\times(300.0/T_g)^{0.50}$ | 25 |

**Table S10.** Ion-electron recombination reactions

| No. | Reaction | Rate coefficients | Ref. |
|---|---|---|---|
| R473 | $e + H_2^+ \rightarrow H + H$ | $3.00\times10^{-08}\times(300.0/T_e)^{0.50}$ | 8 |
| R474 | $e + H_3^+ \rightarrow H + H + H$ | $1.10\times10^{-07}\times(300.0/T_e)^{0.97}$ | 8 |
| R475 | $e + H_3^+ \rightarrow H_2 + H$ | $4.50\times10^{-08}\times(300.0/T_e)^{0.97}$ | 8 |
| R476 | $e + N_2^+ \rightarrow N + N$ | $9.00\times10^{-08}\times(300.0/T_e)^{0.39}$ | 8 |
| R477 | $e + N_2^+ \rightarrow N + N(^2D)$ | $8.10\times10^{-08}\times(300.0/T_e)^{0.39}$ | 8 |
| R478 | $e + N_2^+ \rightarrow N + N(^2P)$ | $9.00\times10^{-09}\times(300.0/T_e)^{0.39}$ | 8 |
| R479 | $e + N_3^+ \rightarrow N_2 + N$ | $2.00\times10^{-07}\times(300.0/T_e)^{0.50}$ | 8 |
| R480 | $e + N_4^+ \rightarrow N_2 + N_2$ | $2.30\times10^{-06}\times(300.0/T_e)^{0.53}$ | 8 |
| R481 | $e + N_2H^+ \rightarrow N_2 + H$ | $7.10\times10^{-07}\times(300.0/T_e)^{0.72}$ | 8 |
| R482 | $e + N^+ \rightarrow N$ | $3.50\times10^{-12}\times(300.0/T_e)^{0.53}\times\exp(3.2/T_e)$ | 25 |
| R483 | $e + H^+ \rightarrow H$ | $3.50\times10^{-12}\times(300.0/T_e)^{0.75}$ | 25 |
| R484 | $e + NH^+ \rightarrow N + H$ | $4.30\times10^{-08}\times(300.0/T_e)^{0.50}$ | 25 |
| R485 | $e + NH_2^+ \rightarrow NH + H$ | $9.21\times10^{-08}\times(300.0/T_e)^{0.79}\times\exp(-17.1/T_e)$ | 25 |
| R486 | $e + NH_2^+ \rightarrow N + H + H$ | $1.78\times10^{-07}\times(300.0/T_e)^{0.80}\times\exp(-17.1/T_e)$ | 25 |
| R487 | $e + NH_3^+ \rightarrow NH + H + H$ | $1.55\times10^{-07}\times(300.0/T_e)^{0.50}$ | 25 |
| R488 | $e + NH_3^+ \rightarrow NH_2 + H$ | $1.55\times10^{-07}\times(300.0/T_e)^{0.50}$ | 25 |
| R489 | $e + NH_4^+ \rightarrow NH_2 + H_2$ | $4.72\times10^{-08}\times(300.0/T_e)^{0.60}$ | 25 |
| R490 | $e + NH_4^+ \rightarrow NH_2 + H + H$ | $3.77\times10^{-08}\times(300.0/T_e)^{0.60}$ | 25 |
| R491 | $e + NH_4^+ \rightarrow NH_3 + H$ | $8.49\times10^{-07}\times(300.0/T_e)^{0.60}$ | 25 |
| R492 | $e + N_2H^+ \rightarrow NH + N$ | $1.92\times10^{-08}\times(300.0/T_e)^{0.84}$ | 25 |
| R493 | $e + H^+ + e \rightarrow H + e$ | $1.29\times10^{-19}\times(300.0/T_e)^{4.50}$ | 23 |

| | | | |
|---|---|---|---|
| R494 | $e + N^+ + e \rightarrow N + e$ | $1.61\times10^{-19}\times(300.0/T_e)^{4.50}$ | 23 |
| R495 | $e + N_2^+ + e \rightarrow N_2 + e$ | $2.08\times10^{-19}\times(300.0/T_e)^{4.50}$ | 23 |
| R496 | $e + H_2^+ + e \rightarrow H_2 + e$ | $1.99\times10^{-19}\times(300.0/T_e)^{4.50}$ | 23 |
| R497 | $e + N_3^+ \rightarrow N + N_2(B3)$ | $4.30\times10^{-07}\times(300.0/T_e)^{0.50}$ | 22 |
| R498 | $e + N_3^+ \rightarrow N + N_2(A3)$ | $4.30\times10^{-07}\times(300.0/T_e)^{0.50}$ | 22 |
| R499 | $e + N^+ + N_2 \rightarrow N + N_2$ | $9.35\times10^{-21}\times(300.0/T_e)^{2.50}$ | 22 |
| R500 | $e + N_2^+ + N_2 \rightarrow N_2(B3) + N_2$ | $9.35\times10^{-21}\times(300.0/T_e)^{2.50}$ | 22 |

## S.4. Summary of input parameters used in the kinetic model

**Table S11.** Key input parameters used in the kinetic model

| Pressure (Bar) | Discharge Power (W) | Temperature (°C) | Pressure (Pa) | Reduced electric field (Td) | Frequency (kHz) | $N_2/H_2$ ratio |
|---|---|---|---|---|---|---|
| 1 | 3, 7, 11, 15 [a] | 132, 182, 210, 226 [b] | 101325 | c | 11 | 1:3 |
| 2 | 3, 7, 11, 15 [a] | 136, 216, 258, 280 [b] | 202650 | c | 11 | 1:3 |
| 3 | 3, 7, 11, 15 [a] | 136, 216, 267, 290 [b] | 303975 | c | 11 | 1:3 |

a, b: The four temperatures correspond directly to the four discharge power values.

c: The reduced electric field was calculated based on the power density.

## S.5. Calculation of the mean reduced electric field

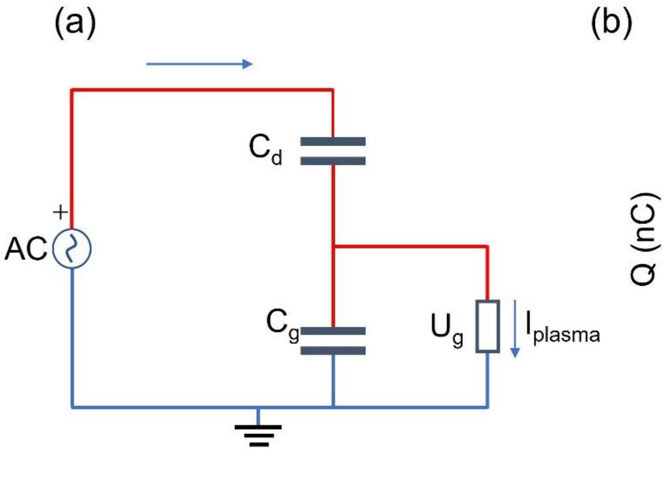


**Figure S2**. (a) Equivalent circuit model of a DBD and (b) the corresponding Q-U plot.

The average electric field ($E$) is defined as:

$$E\left(\text{kV cm}^{-1}\right)=\frac{U_{\text{gap}}\left(\text{kV}\right)}{d_{\text{gap}}\left(\text{cm}\right)} \tag{S1}$$

where $E$ is the average electric field, $U_{\text{gap}}$ is the voltage across the discharge gap, and $d_{\text{gap}}$ is the distance of the discharge gap (1 mm in this study).

The reduced electric field is defined as:

$$E/N\left(\text{Td}\right)=\frac{E\left(\text{V cm}^{-1}\right)}{N\left(\text{cm}^{-3}\right)}\times10^{17} \tag{S2}$$

where $E/N$ is the reduced electric field, and $N$ is the gas number density.

The gas number density is calculated based on the Loschmidt constant:

$$N=\frac{p}{\text{k}_{\text{B}}T}\times10^{-6} \tag{S3}$$

where $p$ is the pressure (Pa), $k_{\text{B}}$ is the Boltzmann constant ($1.38065\times10^{-23}$ J/K) and $T$ is the temperature (K).

## S.6. Reaction pathways for plasma-assisted ammonia synthesis at 1 bar and 2 bar

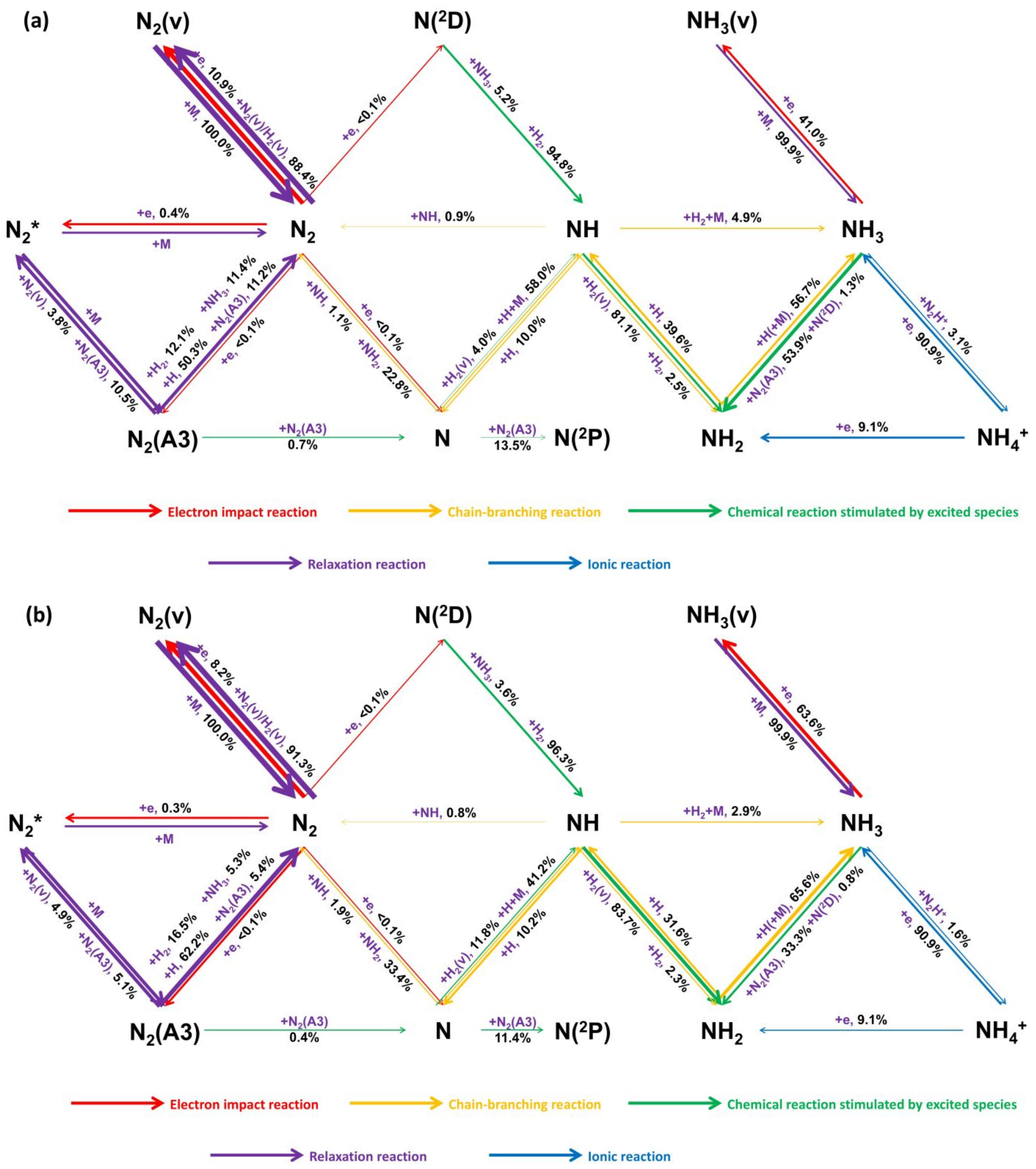


**Figure S3**. Network of plasma-assisted ammonia synthesis in a 0.25 $N_2$/0.75 $H_2$ mixture at 15 W discharge power under the pressures of (a) 1 bar and (b) 2 bar.

## Reference

[1] Itikawa database, www.lxcat.net, retrieved on May 15, 2024.

[2] Morgan database, www.lxcat.net, retrieved on May 15, 2024.

[3] Phelps database, www.lxcat.net, retrieved on May 15, 2024.

[4] Flitti, A.; Pancheshnyi, S. Gas heating in fast pulsed discharges in $N_2$–$O_2$ mixtures. *Eur. Phys. J-Appl. Phys*. **2009**, *45*, 21001, DOI: 10.1051/epjap/2009011

[5] Mao, X.; Chen, Q.; Rousso, A. C.; Chen, T. Y.; Ju, Y. Effects of controlled non-equilibrium excitation on $H_2/O_2$/He ignition using a hybrid repetitive nanosecond and DC discharge. *Combust. Flame* **2019**, *206*, 522-535, DOI: 10.1016/j.combustflame.2019.05.027

[6] Itikawa, Y. Cross sections for electron collisions with ammonia. *J. Phys. Chem. Ref. Data* **2017**, *46*, 043103, DOI: 10.1063/1.5001918

[7] Li, S.; Sun, J.; Gorbanev, Y.; van't Veer, K.; Loenders, B.; Yi, Y.; Kenis, T., Chen, Q., Bogaerts, A. Plasma-assisted dry reforming of $CH_4$: how small amounts of $O_2$ addition can drastically enhance the oxygenate production-experiments and insights from plasma chemical kinetics modeling. *ACS Sustainable Chem. Eng.* **2023**, *11*, 15373-15384, DOI: 10.1021/acssuschemeng.3c04352

[8] Capitelli, M.; Ferreira, C. M.; Gordiets, B. F.; Osipov, A. I. *Plasma kinetics in atmospheric gases*; Springer Series on Atomic, Optical, and Plasma Physics; Springer Berlin Heidelberg: Berlin, Heidelberg, 2000; Vol. 31, DOI: 10.1007/978-3-662-04158-1

[9] Blauer, J.; Nickerson, G. A survey of vibrational relaxation rate data for processes important to $CO_2$-$N_2$-$H_2O$ infrared plume radiation, In *7th Fluid and Plasma Dynamics Conference*, **1974**: 536, DOI: 10.2514/6.1974-536

[10] Faingold, G.; Kalitzky, O.; Lefkowitz, J. K. Plasma reforming for enhanced ammonia-air ignition: a numerical study. *Fuel Communications*, **2022**, *12*, 100070, DOI: 10.1016/j.jfueco.2022.100070

[11] Zhong, H.; Mao, X.; Liu, N.; Wang, Z.; Ombrello, T.; Ju, Y. Understanding non-equilibrium $N_2O/NO_x$ chemistry in plasma-assisted low-temperature $NH_3$ oxidation. *Combust. Flame* **2023**, *256*, 112948, DOI: 10.1016/j.combustflame.2023.112948

[12] Kable, S. H.; Knight, A. E. W. Semiempirical model of vibrational relaxation for estimating absolute rate coefficients. *J. Phys. Chem. A* **2003**, *107*, 10813-10825, DOI: 10.1021/jp035516u

[13] Hovis, F. E.; Moore, C. B. Vibrational relaxation of $NH_3$(v2). *J. Chem. Phys*. **1978**, *69*, 4947, DOI: 10.1063/1.436482

[14] Hong, J.; Pancheshnyi, S.; Tam, E.; Lowke, J. J.; Prawer, S.; Murphy, A. B. Kinetic modelling of $NH_3$ production

in $N_2$–$H_2$ non-equilibrium atmospheric-pressure plasma catalysis. *J. Phys. D: Appl. Phys.* **2017**, *50*, 154005, DOI: 10.1088/1361-6463/aa6229

[15] Gordiets, B.; Ferreira, C. M.; Pinheiro, M. J.; Ricard, A. Self-consistent kinetic model of low-pressure-flowing discharges: I. Volume processes. *Plasma Sources Sci. Technol*. **1998**, *7*, 363, DOI: 10.1088/0963-0252/7/3/015

[16] Mao, X.; Zhong, H.; Liu, N.; Wang, Z.; Ju, Y. Ignition enhancement and $NO_x$ formation of $NH_3$/air mixtures by non-equilibrium plasma discharge. *Combust. Flame* **2024**, *259*, 113140, DOI: 10.1016/j.combustflame.2023.113140

[17] Fridman, A. *Plasma chemistry*, Cambridge University Press: Cambridge, UK, 2008.

[18] Torr, M. R.; Torr, D. G.; Richards, P. G. N(2P) in the dayglow: measurement and theory. *Geophys. Res. Lett*. **1993**, *20*, 531-534, DOI: 10.1029/92GL02180

[19] Dutuit, O.; Carrasco, N.; Thissen, R. Critical review of N, $N^+$, $N_2^+$, $N^{++}$, and $N_2^{++}$ main production processes and reactions of relevance to Titan's atmosphere. *The Astrophysical Journal Supplement Series* **2013**, *204*, 20, DOI: 10.1088/0067-0049/204/2/20

[20] Tsyganov, D.; Pancheshnyi, S. Simulation of N-atom production in dielectric-barrier discharge in nitrogen at atmospheric pressure. *Plasma Sources Sci. Technol.* **2012**, *21*, 065010, DOI: 10.1088/0963-0252/21/6/065010

[21] Kobayashi, S.; Bonaventura, Z.; Tholin, F.; Popov, N. A.; Bourdon, A. Study of nanosecond discharges in $H_2$–air mixtures at atmospheric pressure for plasma assisted combustion applications. *Plasma Sources Sci. Technol*. **2017**, *26*, 075004, DOI: 10.1088/1361-6595/aa729a

[22] Starikovskiy, A. Mechanism of plasma-assisted ignition for $H_2$ and $C_1$-$C_5$ hydrocarbons. In *55th AIAA Aerospace Sciences Meeting*. ***2017***: 1977, DOI: 10.2514/6.2017-1977

[23] DeFilippo, A. C.; Chen, J. Y. Modeling plasma-assisted methane–air ignition using pre-calculated electron impact reaction rates. *Combust. Flame* **2016**, *172*, 38-48, DOI: 10.1016/j.combustflame.2016.07.005

[24] Herron, J. T. Evaluated chemical kinetics data for reactions of N($^2$D), N($^2$P), and $N_2(A3\Sigma_u^+)$ in the gas phase. *J. Phys. Chem. Ref. Data* **1999**, *28*, 1453-1483, DOI: 10.1063/1.556043

[25] The UMIST Database for Astrochemistry, udfa.ajmarkwick.net

[26] Liu, D. X.; Iza, F.; Wang, X. H.; Ma, Z. Z.; Rong. M. Z.; Kong M. G. A theoretical insight into low-temperature atmospheric-pressure He+$H_2$ plasmas. *Plasma Sources Sci. Technol.* **2013**, *22*, 055016, DOI: 10.1088/0963-0252/22/5/055016

[27] Manion, J. A.; Huie, R. E.; Levin, R. D.; Burgess, Jr. D. R.; Orkin, V. L. *NIST Chemical Kinetics Database*, NIST Standard Reference Database 17, Version 7.0 (Web Version), Release 1.6.8, Data version 2015.09, National Institute of Standards and Technology: Gaithersburg, Maryland, 20899-8320. https://kinetics.nist.gov/
